# Oncoplastic breast consortium recommendations for mastectomy and whole breast reconstruction in the setting of post-mastectomy radiation therapy

**DOI:** 10.1016/j.breast.2022.03.008

**Published:** 2022-03-18

**Authors:** Walter Paul Weber, Jane Shaw, Andrea Pusic, Lynda Wyld, Monica Morrow, Tari King, Zoltán Mátrai, Jörg Heil, Florian Fitzal, Shelley Potter, Isabel T. Rubio, Maria-Joao Cardoso, Oreste Davide Gentilini, Viviana Galimberti, Virgilio Sacchini, Emiel J.T. Rutgers, John Benson, Tanir M. Allweis, Martin Haug, Regis R. Paulinelli, Tibor Kovacs, Yves Harder, Bahadir M. Gulluoglu, Eduardo Gonzalez, Andree Faridi, Elisabeth Elder, Peter Dubsky, Jens-Uwe Blohmer, Vesna Bjelic-Radisic, Mitchel Barry, Susanne Dieroff Hay, Kimberly Bowles, James French, Roland Reitsamer, Rupert Koller, Peter Schrenk, Daniela Kauer-Dorner, Jorge Biazus, Fabricio Brenelli, Jaime Letzkus, Ramon Saccilotto, Sarianna Joukainen, Susanna Kauhanen, Ulla Karhunen-Enckell, Juergen Hoffmann, Ulrich Kneser, Thorsten Kühn, Michalis Kontos, Ekaterini Christina Tampaki, Moshe Carmon, Tal Hadar, Giuseppe Catanuto, Carlos A. Garcia-Etienne, Linetta Koppert, Pedro F. Gouveia, Jakob Lagergren, Tor Svensjö, Nadia Maggi, Elisabeth A. Kappos, Fabienne D. Schwab, Liliana Castrezana, Daniel Steffens, Janna Krol, Christoph Tausch, Andreas Günthert, Michael Knauer, Maria C. Katapodi, Susanne Bucher, Nik Hauser, Christian Kurzeder, Rosine Mucklow, Pelagia G. Tsoutsou, Atakan Sezer, Güldeniz Karadeniz Çakmak, Hasan Karanlik, Patricia Fairbrother, Laszlo Romics, Giacomo Montagna, Cicero Urban, Melanie Walker, Silvia C. Formenti, Guenther Gruber, Frank Zimmermann, Daniel Rudolf Zwahlen, Sherko Kuemmel, Mahmoud El-Tamer, Marie Jeanne Vrancken Peeters, Orit Kaidar-Person, Michael Gnant, Philip Poortmans, Jana de Boniface

**Affiliations:** a Breast Center, University Hospital Basel, Basel, Switzerland; b University of Basel, Basel, Switzerland; c Patient Advocacy Group, Oncoplastic Breast Consortium, Basel, Switzerland; d Brigham and Women's/Dana Farber Cancer Center, USA; e Department of Oncology and Metabolism, University of Sheffield, Sheffield, UK; f Breast Surgery Service, Memorial Sloan Kettering Cancer Center, New York, NY, USA; g Department of Surgery, Brigham and Women's Hospital / Dana Farber Cancer Institute, USA; h Department of Breast and Sarcoma Surgery, National Institute of Oncology, Budapest, Hungary; i Department of Obstetrics and Gynecology, University of Heidelberg, Medical School, Heidelberg, Germany; j Department of Surgery and Comprehensive Cancer Center, Medical University of Vienna, Vienna, Austria; k Bristol Centre for Surgical Research, Bristol Medical School, University of Bristol, Clifton, Bristol, UK; l Breast Surgical Oncology, Clinica Universidad de Navarra, Madrid, Spain; m Breast Unit, Champalimaud Clinical Centre, Champalimaud Foundation, And Nova Medical School, Lisbon, Portugal; n IRCCS Ospedale San Raffaele, Milan, Italy; o Istituto Europeo di Oncologia, IRCCS, Milan, Italy; p Department of Surgery, The Netherlands Cancer Institute, Antoni van Leeuwenhoek Hospital, Amsterdam, the Netherlands; q Cambridge Breast Unit, Addenbrooke's Hospital Cambridge, Cambridge, UK; r Cambridge Breast Unit, Cambridge University Hospitals NHS Foundation TRUST, School of Medicine, Anglia Ruskin University, Cambridge, UK; s Hadassah Medical Center & Faculty of Medicine, Hebrew University, Jerusalem, Israel; t Breast Center and Department of Plastic, Reconstructive, Aesthetic and Handsurgery University Hospital Basel, University of Basel, Basel, Switzerland; u Federal University of Goiás, Araújo Jorge Hospital, Goiás Anti-Cancer Association, Goiás, Brazil; v Jiahui Internatioonal Hospital Shanghai, China; w Guy's and St. Thomas' NHS Foundation Trust London, UK; x Department of Plastic, Reconstructive and Aesthetic Surgery, Ospedale Regionale di Lugano, Ente Ospedaliero Cantonale (EOC), Lugano, Switzerland; y Faculty of Biomedical Sciences, Università Della Svizzera Italiana, Lugano, Switzerland; z Marmara University School of Medicine, Istanbul, Turkey; aa Departament of Mastology, Breast Unit- Instituto de Oncología Angel H Roffo, Buenos Aires Univesity. Buenos Aires, Argentina; ab Department of Senology/Breast Center, University Hospital Bonn, Germany; ac Westmead Breast Cancer Institute, Westmead Hospital, University of Sydney, Australia; ad Breast Center, Hirslanden Clinic St. Anna, Lucerne, Switzerland; ae Department of Gynecology and Breast Center, Charité University Hospital, Berlin, Germany; af Breast Unit, Helios University Hospital, University Witten/Herdecke, Wuppertal, Germany; ag Mater Misericordiae University Hospital, Dublin, Ireland; ah Patient Advocacy Group, Oncoplastic Breast Consortium, President, the Swedish Breast Cancer Association, Stockholm, Sweden; ai Patient Advocacy Group, Oncoplastic Breast Consortium, Not Putting on A Shirt, Pittsburgh, USA; aj Breast Center Salzburg, University Clinic Salzburg, Paracelsus Medical University Salzburg, Salzburg, Austria; ak Department of Plastic, Aesthetic and Reconstructive Surgery, Vienna Health Services, Clinic Landstrasse and Clinic Ottakring, Vienna, Austria; al Breast Cancer Center, Kepler University Hospital, Linz, Austria; am Department of Radiooncology, University Hospital Vienna, Austria; an Division of Breast Surgery, Universidade Federal Do Rio Grande Do Sul (UFRGS), Porto Alegre, Brazil; ao Breast Oncology Division, University of Campinas, Campinas, São Paulo, Brazil; ap San Borja Arriaran Clinical Hospital, University of Chile, Chile; aq Kuopio University Hospital, Kuopio, Finland; ar Department of Plastic Surgery, University of Helsinki and Helsinki University Hospital, Helsinki, Finland; as Tampere University Hospital, Department of Surgery and Tays Cancer Center, Tampere, Finland; at Breast Center, University Hospital Düsseldorf, Düsseldorf, Germany; au Department of Hand, Plastic and Reconstructive Surgery - Burn Center, BG Trauma Center Ludwigshafen/Rhine, Hand and Plastic Surgery, University Heidelberg, Heidelberg, Germany; av Interdisciplinary Breast Center, Klinikum Esslingen, Germany; aw National and Kapodistrian University of Athens, Greece; ax Department of Plastic, Reconstructive Surgeryand Burn Unit, KAT Athens Hospital and Trauma Center, Athens, Greece; ay Tel Aviv Soraski Medical Center, Israel; az Multidisciplinary Breast Unit, Azienda Ospedaliera Cannizzaro, Catania, Italy; ba Breast Unit, Ospedale Santa Chiara, Trento, Italy; bb Department of Surgery, Erasmus MC Cancer Institute, Rotterdam, the Netherlands; bc Department of Surgery, Capio St Goran's Hospital, Stockholm, Sweden; bd Department of Molecular Medicine and Surgery, Karolinska Institutet, Stockholm, Sweden; be Department of Surgery, Central Hospital, Kristianstad, Sweden; bf Breast Center Zurich, Zurich, Switzerland; bg Gyn-zentrum, Luzern, Switzerland; bh Breast Center Eastern Switzerland, St. Gallen, Switzerland; bi Breast Center, Lucerne Cantonal Hospital, Lucerne, Switzerland; bj Breast Center, Hirslanden Clinic Aarau, Aarau, Frauenarztzentrum Aargau AG, Baden, Switzerland; bk University Hospital Geneva, University of Geneva, Faculty of Medicine, Geneva, Switzerland; bl Department of Surgery, Trakya University Medical School Hospital, Turkey; bm Department of Surgery, The School of Medicine, Zonguldak Bulent Ecevit University, Zonguldak, Turkey; bn Istanbul University Institute of Oncology, Turkey; bo Patient Advocacy Group, Oncoplastic Breast Consortium, Breakthrough Breast Cancer, Association Breast Surgery UKBCC, Kedleston, UK; bp Department of Surgery, New Victoria Hospital, Glasgow, UK; bq Breast Unit, Hospital Nossa Senhora Das Graças, Curitiba, Brazil; br Breast Endocrine and General Surgery Unit, The Alfred, Melbourne, Australia; bs Breast Surgeons of Australia and New Zealand (BreastSurgANZ), Australia; bt Department of Radiation Oncology and Meyer Cancer Center, Weill Cornell Medicine, USA; bu Institute for Radiotherapy, Klinik Hirslanden, 8032, Zurich, Switzerland; bv University of Berne, 3000, Bern, Switzerland; bw Clinic of Radiation Oncology, University Hospital Basel, Basel, Switzerland; bx Department of Radiation Oncology, Cantonal Hospital of Winterthur, Winterthur, Switzerland; by Breast Unit, Kliniken Essen-Mitte, Germany; bz Department of Surgical Oncology Netherlands Cancer Institute, Antoni van Leeuwenhoek & Amsterdam University Medical Center, Netherlands; ca Breast Radiation Therapy Unit, Sheba Tel Hashomer, Ramat Gan, Israel; cb Sackler School of Medicine, Tel-Aviv University, Tel-Aviv, Israel; cc Comprehensive Cancer Center, Medical University of Vienna, Vienna, Austria; cd Iridium Netwerk and University of Antwerp, Wilrijk-Antwerpen, Belgium; ce Department of Surgery, Capio St Göran's Hospital, Stockholm, Sweden

**Keywords:** Breast cancer, Post-mastectomy radiotherapy, Nipple-sparing mastectomy, Implant-based breast reconstruction, Autologous breast reconstruction

## Abstract

**Aim:**

Demand for nipple- and skin- sparing mastectomy (NSM/SSM) with immediate breast reconstruction (BR) has increased at the same time as indications for post-mastectomy radiation therapy (PMRT) have broadened. The aim of the Oncoplastic Breast Consortium initiative was to address relevant questions arising with this clinically challenging scenario.

**Methods:**

A large global panel of oncologic, oncoplastic and reconstructive breast surgeons, patient advocates and radiation oncologists developed recommendations for clinical practice in an iterative process based on the principles of Delphi methodology.

**Results:**

The panel agreed that surgical technique for NSM/SSM should not be formally modified when PMRT is planned with preference for autologous over implant-based BR due to lower risk of long-term complications and support for immediate and delayed-immediate reconstructive approaches. Nevertheless, it was strongly believed that PMRT is not an absolute contraindication for implant-based or other types of BR, but no specific recommendations regarding implant positioning, use of mesh or timing were made due to absence of high-quality evidence. The panel endorsed use of patient-reported outcomes in clinical practice. It was acknowledged that the shape and size of reconstructed breasts can hinder radiotherapy planning and attention to details of PMRT techniques is important in determining aesthetic outcomes after immediate BR.

**Conclusions:**

The panel endorsed the need for prospective, ideally randomised phase III studies and for surgical and radiation oncology teams to work together for determination of optimal sequencing and techniques for PMRT for each patient in the context of BR

## Highlights

• Autologous breast reconstruction is increasingly preferred over implants in the setting of radiation therapy.
• Use of patient-reported outcomes is endorsed.
• Shape and size of reconstructed breasts can hinder radiotherapy planning.
• There is a need for randomised phase III trials.

## 1 Introduction

Selection criteria for nipple- or skin-sparing mastectomy (NSM and SSM respectively) in conjunction with immediate breast reconstruction (BR) have become less stringent with an increase in proportion of patients potentially eligible for breast conserving therapy undergoing mastectomy and BR [1,2]. A parallel trend has been broadening of the indications for post-mastectomy radiation therapy (PMRT) that is often combined with nodal irradiation for low volume nodal disease [[3], [4], [5], [6], [7], [8]]. Hence, there is dual consideration of both BR and PMRT for many patients who undergo mastectomy for surgical treatment of breast cancer [9,10]. PMRT increases risk of complications and diminishes aesthetic outcomes and quality of life (QoL) following BR, especially when implant-based [[11], [12], [13]]. The 2018 OPBC consensus conference revealed major heterogeneity in BR practice in the context of planned PMRT with a majority of the panel agreeing that type and timing of BR in this setting should be standardized [14]. The 2019 OPBC consensus conference ranked type and timing of BR in the setting of PMRT as the two most important knowledge gaps in the wider field of BR [15]. This year's OPBC consensus conference therefore systematically addressed relevant questions pertaining to type and timing of BR when PMRT is planned and provided expert recommendations for clinical practice.

## 2 Material and methods

### 2.1 2021 OPBC expert panel

The OPBC was founded in March 2017 as a global non-profit organization and comprises a membership of 616 oncologic, oncoplastic and reconstructive breast surgeons and 38 patient advocates from 79 countries at the time of manuscript writing. The OPBC is committed to bringing safe and effective oncoplastic breast surgery to routine patient care, namely oncoplastic breast conserving surgery, NSM/SSM with immediate BR and aesthetic flat closure after conventional mastectomy. The global 2021 OPBC expert panel was selected by evident expertise in breast cancer management with a practice primarily dedicated to breast cancer. Panellists originated from 22 countries and included 68 oncologic, oncoplastic and plastic breast surgeons from private, public, community and academic settings, six patients with international renown as patient advocates along with nine radiation oncologists with robust scientific credentials and international standing (appendix B.3.1–2). Finally, 52 non-panel OPBC members attended the conference and performed live audience voting, which was displayed separately to panel voting (appendix B3.3.).

### 2.2 Search strategy and selection criteria

We purposefully refrained from performing a systematic literature search as a basis for questionnaire development in order for the OPBC to identify and address questions relevant to current clinical practice irrespective of available evidence to inform treatment. Nonetheless, in support of these aims, two members of staff (Elisabeth Kappos and Nadia Maggi) independently performed specific searches in PubMed, MEDLINE, Embase and the Cochrane Central Register of Controlled Trials (CENTRAL) from 2000 to 2021 (search terms “mastectomy, subcutaneous” OR “mastectomy” AND “subcutaneous” OR “subcutaneous mastectomy” OR “nipple” AND “sparing” AND “mastectomy” OR “nipple sparing mastectomy” OR “breast reconstruction” OR “whole-breast reconstruction” OR “breast reconstructive surgery” OR autologous breast reconstruction” OR “implant-based breast reconstruction” OR “post-mastectomy radiotherapy OR “irradiation” OR “radiotherapy” OR “breast reconstruction algorithm” OR “PMRT reconstruction” OR “PMRT breast reconstruction” OR “breast reconstruction algorithm radiation” OR “breast reconstruction” AND “radiation”). Their review of all abstracts and full texts of relevant articles was used to finalize the questionnaire and helped the chairs and moderators to prepare for the consensus conference. Questions, answers and content of discussions were placed in context with published evidence in the form of this report.

### 2.3 Development of questionnaire for pre-voting

The iterative process in question development, pre-voting, presentation of results, discussion, live re-voting and development of phraseology for recommendation outcomes followed a modified Delphi methodology. The predefined protocol was published on the OPBC website on June 08, 2021 (appendix A) [16]. The protocol pre-specified the identification of questions to include, as follows: Those questions from the OPBC 2018 conference that reported disagreement among experts on NSM/SSM and immediate BR were included with the two co-chairs adding key questions based on their expert opinion. This preliminary set of questions was amended by expert representatives based on the specific literature search. At that point in time, the list was sent for review to the entire OPBC community as well as nine radiation oncologists. The chairs adjusted these questions according to feedback and finalized the list by iterative consultation with the panellists over the months preceding the conference (appendix C).

The iterative voting process started with pre-voting, which also allowed participation of conference non-attenders, provided opportunity to prepare the agenda for live voting that focused on areas of controversy, and served as back-up in the event of technical failure during live conference voting. Results of pre-voting were revealed to panel and audience for the first time during the conference thereby promoting spontaneous discussion.

### 2.4 Consensus conference with live voting

The 2021 OPBC consensus conference on September 02, 2021 was held virtually using online video conferencing software (Zoom by Zoom Video Communications, Inc). This platform provided separate rooms for the OPBC panel and OPBC members who registered for audience participation. Three panel members presented their respective views as plastic surgeon, oncoplastic surgeon and radiation oncologist with subsequent structured discussion. In the second half, outcomes of pre-voting were presented, followed by live voting by both panellists and audience in case of controversy identified from pre-voting and whenever pre-voting results were challenged or demanded reinforcement. In addition, the customized live voting platform allowed questions to be devised ad hoc based on panel discussion. Results of live voting were displayed separately for the OPBC panel versus audience.

### 2.5 Final questionnaire

**Fig. 1:**
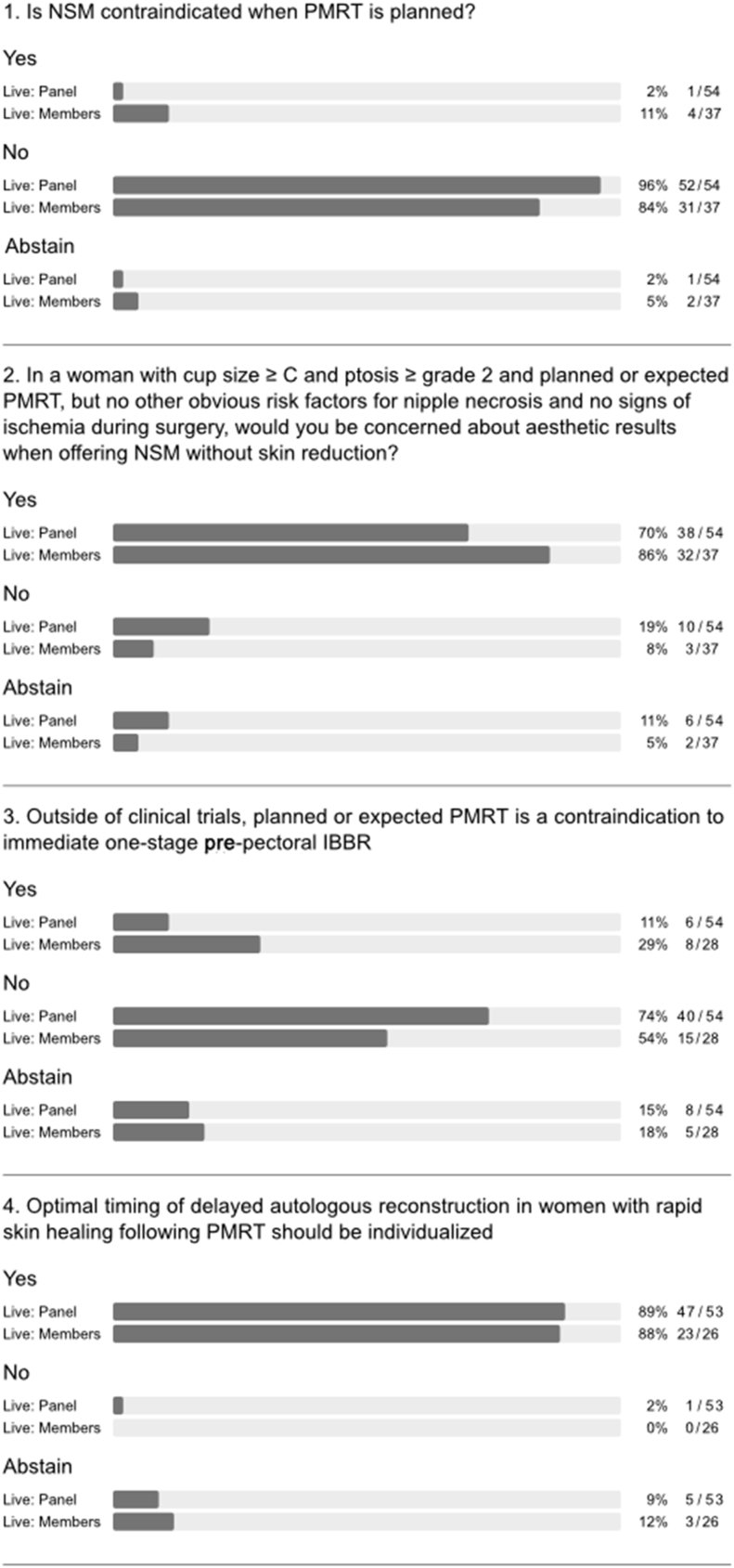

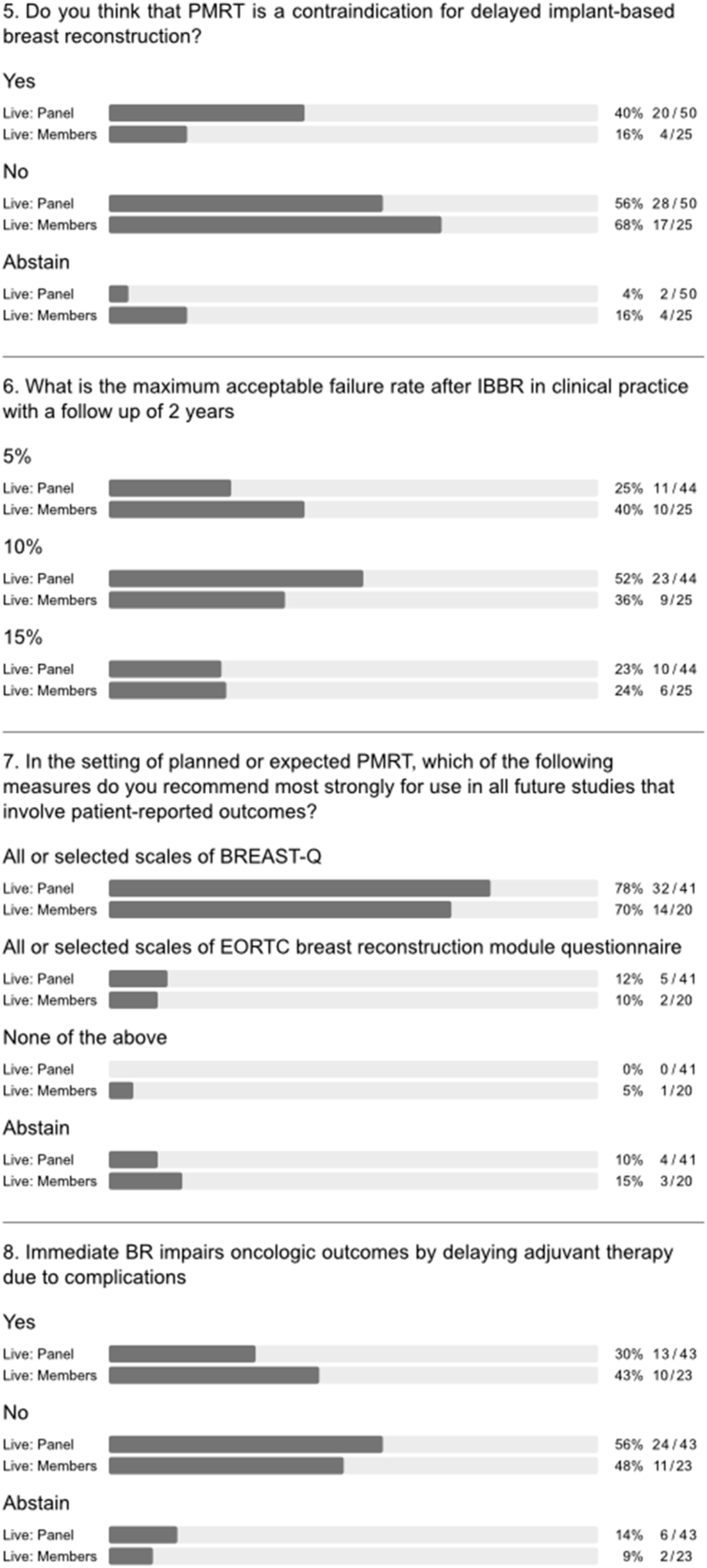
**Questions developed or adjusted ad hoc during consensus conference** Abbreviations used in questionnaire: NSM (nipple-sparing mastectomy), PMRT (post-mastectomy radiotheraphy), BR (Breast reconstruction), IBRR (implant-based breast reconstruction)

The final questionnaire comprised a total of 66 questions and subquestions in nine categories. Eight questions were newly formulated or adjusted ad hoc during the conference based on the discussion (Fig. 1); live re-voting was performed for five questions whilst no live re-voting was recommended for the remaining 53 questions with results of pre-voting being reported. The answers yes, no or abstain applied to 54 statements or questions whilst the single most appropriate answer from a list of options applied in 12. Simple majority was defined by agreement among 51–75% of participants and consensus by agreement above 75%. Abstaining was recommended when panel members had any conflict of interest or considered the question not to be clear, outside their expertise, or the correct answer was missing. All abstentions were reported and included in percentages unless otherwise stated.

### 2.6 Report

Questions, answers and content of discussions were placed in context with current published evidence in the form of this report. Specific details of the literature search were scrutinised by chairs and expert representatives with inclusion of additional references cited in articles identified through searches of personal files. The report was circulated among all panellists as part of an iterative process until agreement was reached on the precise wording of each question such that this reflected the strength of panel support for each recommendation. Voting results are shown graphically and as exact numbers.

## 3 Results and discussion

Consensus agreement was reached on 20 questions, majority agreement on 21, no consensus and no majority on a further 21 with the strength of agreement differing between panellists and members in four questions (Fig. 1, Fig. 5, Fig. 7, and appendix figure E1). A total of 73 panellists completed the pre-voting questionnaire; 59 panellists and 52 members participated in live conference voting.

### 3.1 Nipple- and skin sparing mastectomy

**Fig. 2:**
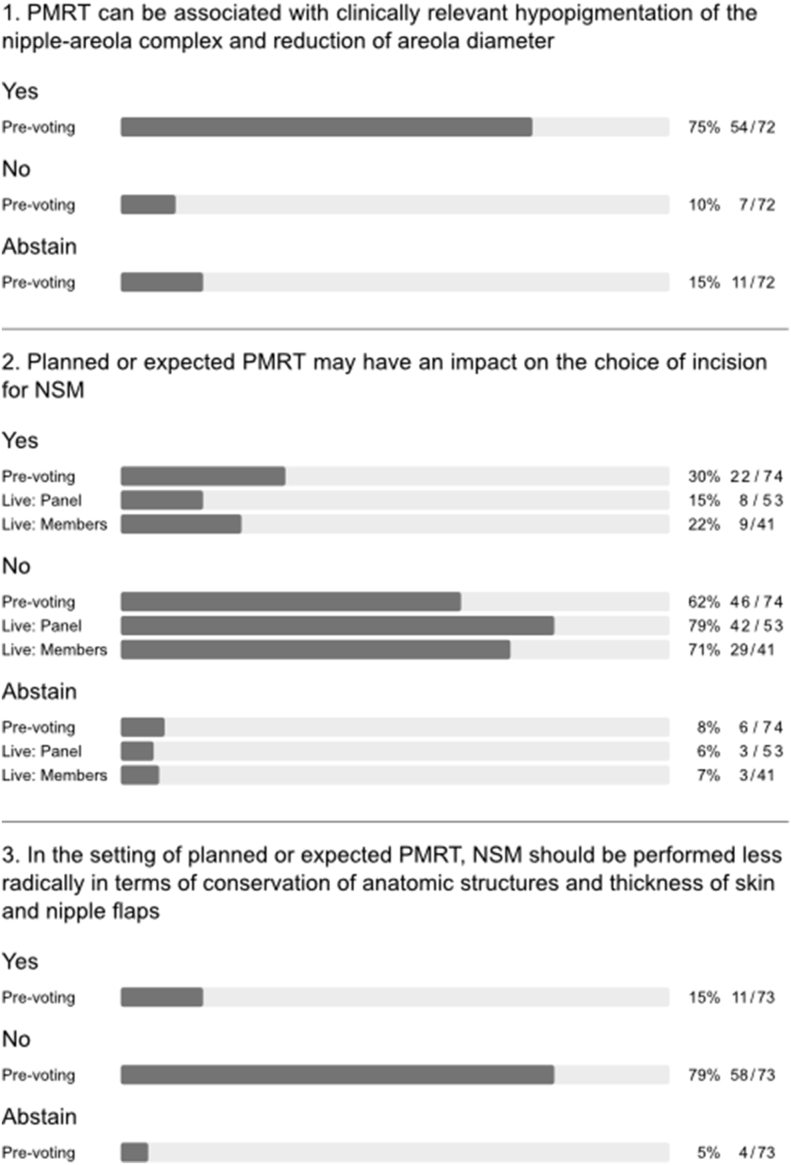
Questions on nipple- and skin-sparing mastectomy.

Both OPBC panel and audience stated with strong consensus that NSM is not contraindicated when PMRT is planned (question (q) 1, Fig. 1). There was broad agreement that PMRT can be associated with hypopigmentation and shrinkage of the nipple-areola complex (NAC; q1, Fig. 2). A majority of both panel and audience felt that planned or anticipated PMRT should not usually have any impact on choice of skin incision (q2, Fig. 2). However, the panel acknowledged consistent observations in the literature that type of incision is linked to risk of complications and noted that the 2018 OPBC panel considered location of incision to be a risk factor for severe mastectomy flap necrosis [14,17,18]. There was no agreement regarding the use of NSM in conjunction with skin reduction and/or fashioning of NAC pedicles or free nipple grafting for large ptotic breasts (q1a-d, appendix figure E1); a strong majority of both panel and audience raised concerns about aesthetic results when offering NSM to this group of patients without skin reduction (q2, Fig. 1). Importantly, there was panel consensus that attempts to perform a less radical form of NSM when PMRT is planned should be avoided (q3, Fig. 2). Thickness of mastectomy flaps cannot be surgically modulated based on need for PMRT – this is pre-determined by patient anatomy and depth of the oncologic plane [19].

### 3.2 Type of breast reconstruction

**Fig. 3:**
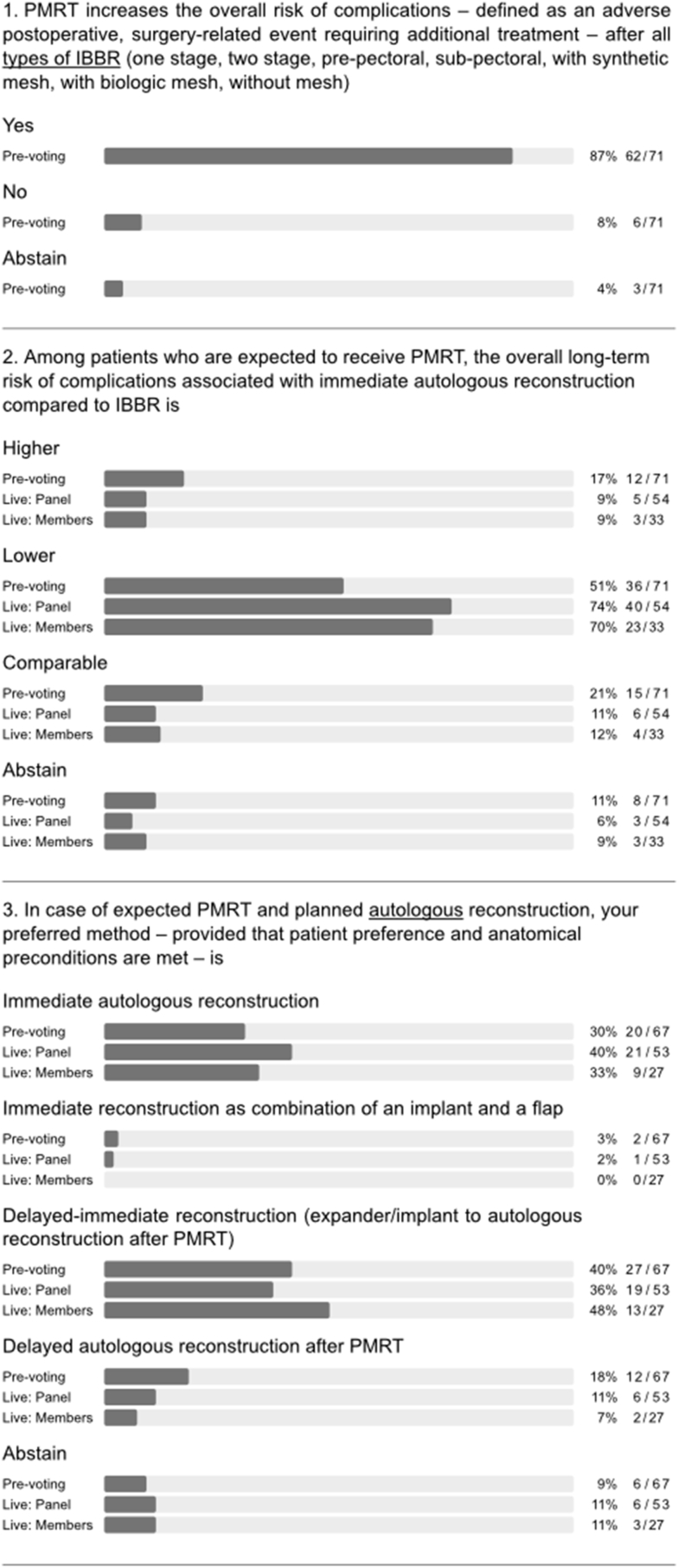

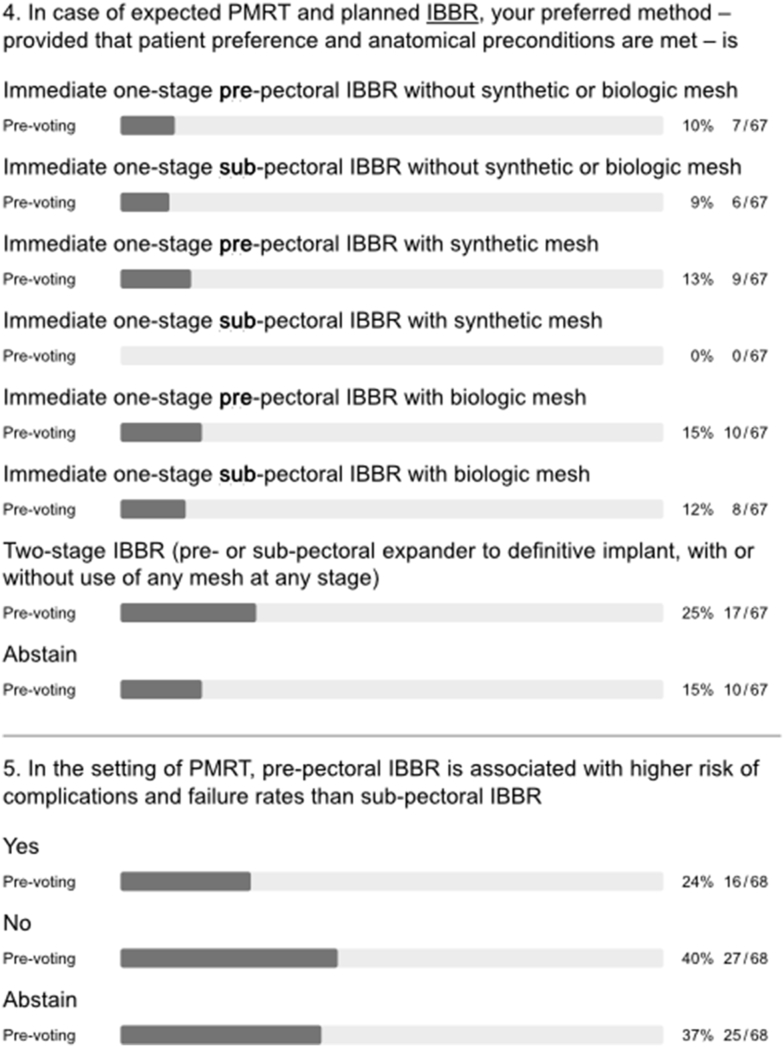
Type of breast reconstruction.

There was general consensus that PMRT increases the risk of complications following all types of implant-based BR (q1, Fig. 3) in agreement with the published literature [11,13,20]. Interestingly, a majority also held the view that PMRT significantly increases complication risk after immediate autologous BR despite results of the Mastectomy Reconstruction Outcomes Consortium (MROC) study (q2a-e, appendix figure E1) [13]. During the conference, one of the authors of this prospective multicentre cohort study discussed the report, which compared complications and patient-reported outcomes (PROs) for 622 irradiated and 1625 non-irradiated patients undergoing implant-based and autologous BR between 2012 and 2015. Among patients who underwent autologous BR, PMRT did not increase the risk of complications. Among patients who received PMRT, autologous reconstruction was associated with lower risk of complications than was implant-based BR (OR = 0.47, 95% CI = 0.27 to 0.82, p = 0.007) and a higher BREAST-Q satisfaction with breasts score (63.5 vs 47.7; p = 0.002). The measurable impact of PMRT on QoL after implant-based BR was confirmed by another large survey of breast cancer survivors [21]. Following extensive discussion of these data, a strong majority of both panel and audience agreed that the overall long-term risk of complications in the setting of PMRT is lower after immediate autologous reconstruction compared to implant-based BR (q2, Fig. 3). When asked about timing of autologous BR in the setting of PMRT, the panel clearly favoured immediate (direct to autologous BR) or delayed-immediate (immediate use of temporary implant or expander until delayed autologous BR) over fully delayed autologous reconstruction (Q3, Fig. 3). In general, autologous BR options were preferred over all implant-based BR options in the setting of PMRT (q4, appendix figure E1). Nevertheless, the panel strongly felt that planned or anticipated PMRT is not an absolute contraindication for any type of BR (q3a-h, appendix figure E1).

Major heterogeneity in clinical practice was evident for implant-based BR in the setting of PMRT. No majority or consensus agreement was reached in terms of recommendations for type, timing, implant position, or use of mesh (q4, Fig. 3). Furthermore, panellists disagreed on whether pre-pectoral implant-based BR is associated with a higher risk of complications and failure rates than sub-pectoral implant-based BR in the context of PMRT (q5, Fig. 3). A majority of the panel considered the use of immediate one-stage pre-pectoral implant-based BR to be compatible with PMRT whilst more of the audience displayed uncertainty on this point (q3, Fig. 1).

### 3.3 Timing of breast reconstruction

**Fig. 4:**
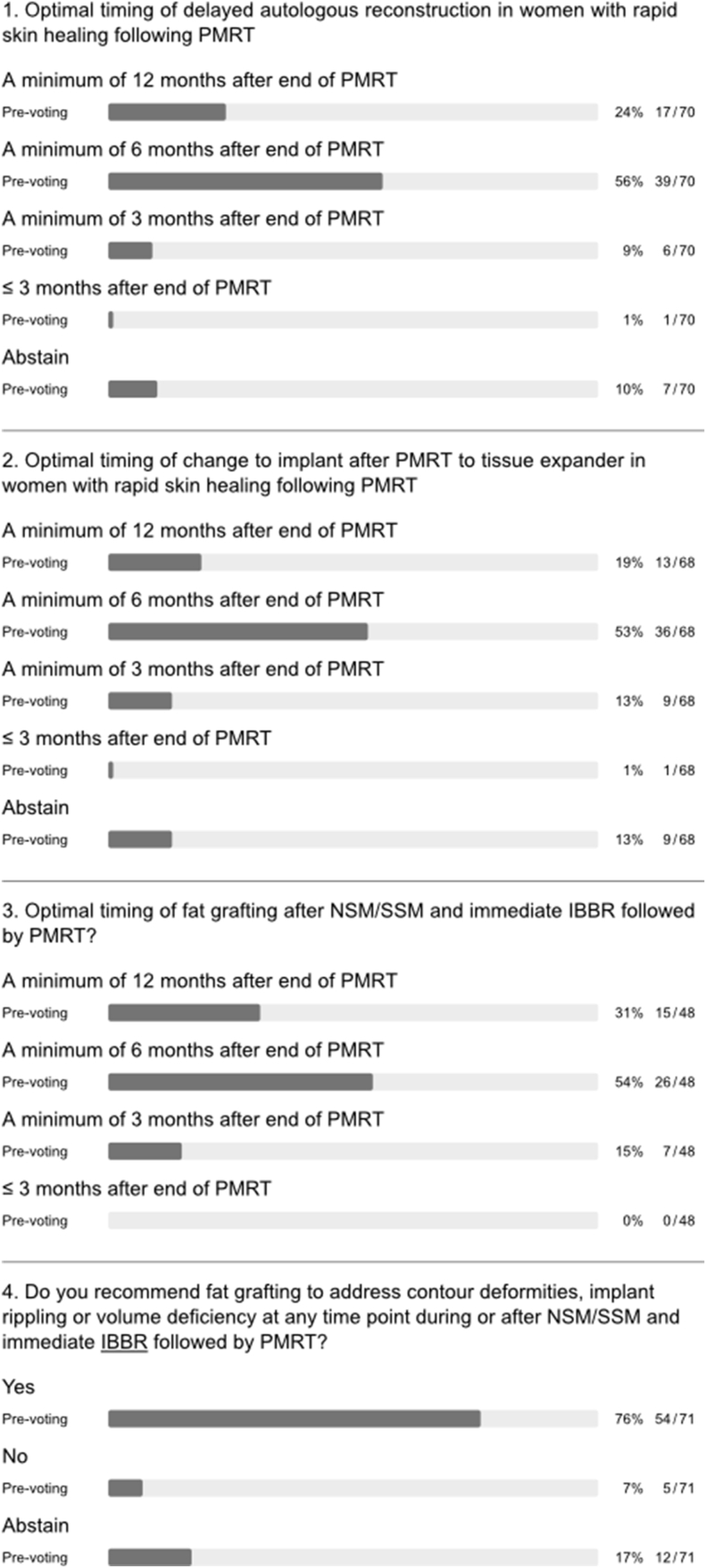

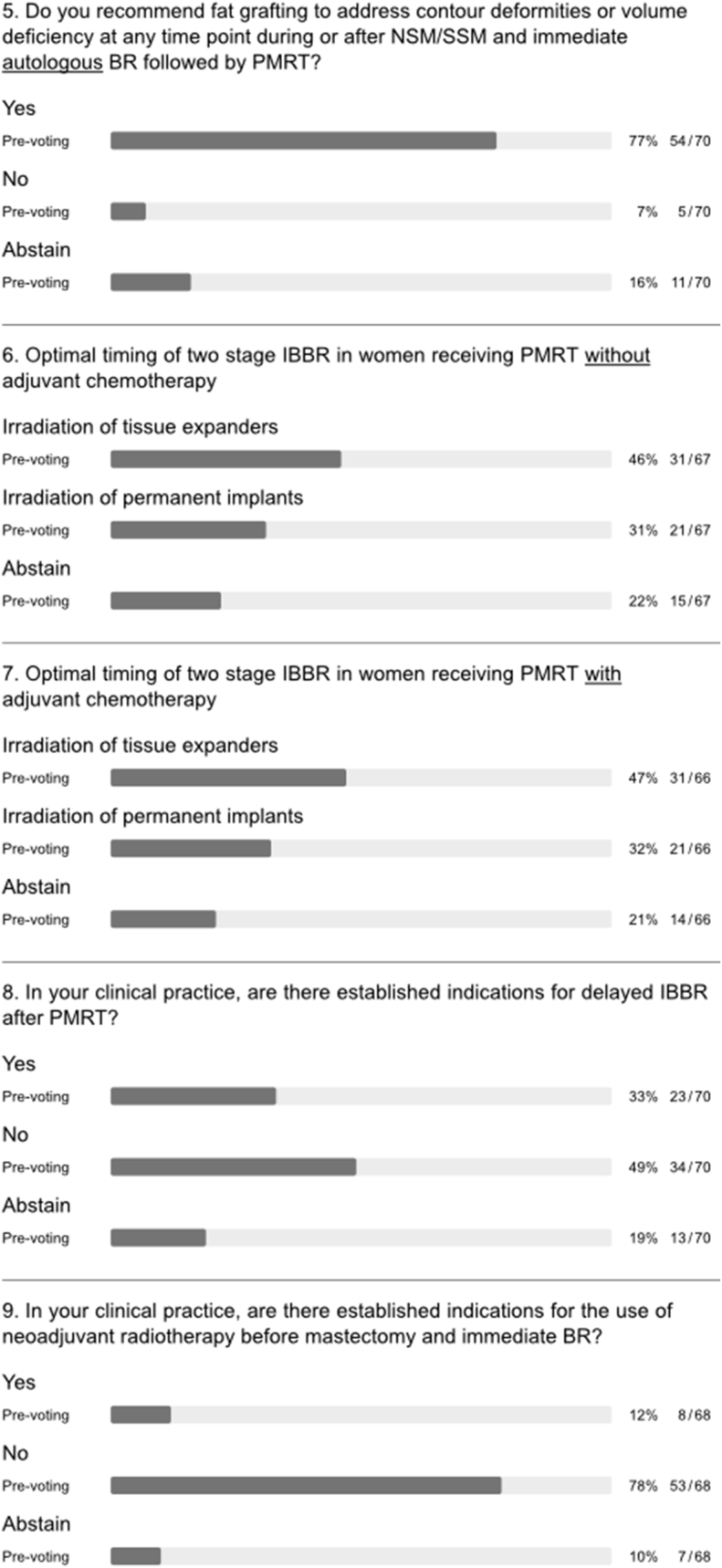
Timing of breast reconstruction.

A strong panel majority recommended waiting for a minimum of 6–12 months after initial surgery in the setting of PMRT, both before delayed autologous BR and exchange of tissue expander for a permanent implant (q1 and 2, Fig. 4). During discussion, the panel emphasized that the optimal timing of delayed autologous reconstruction should be individualized (q4, Fig. 1) and also recommended waiting for 6–12 months before performing fat grafting. The latter was recommended as a method for improving outcomes after both autologous and implant-based BR (q3-5, Fig. 4). The panel was divided on the issue of irradiation of the tissue expander or the permanent implant in two-stage implant-based BR (with or without adjuvant chemotherapy; q6 and 7, Fig. 4). Indeed, several large series have shown that favourable outcomes can be achieved with implant-based BR in the context of radiotherapy using either timing strategy for the two-stage approach [22,23]. Although the panel acknowledged that there are no specific indications for neoadjuvant radiotherapy in routine clinical practice, there was a difference of opinion on delayed implant-based BR after PMRT (q8 and 9, Fig. 4). A majority of panellists who perform delayed implant-based BR discouraged use of highly cohesive implants, smooth implants, polyurethane implants and synthetic mesh in efforts to reduce complications, while advocating use of biologic mesh and fat grafting for purposes of delayed IBBR (q6a-e and h, appendix figure E1). Nonetheless, there was no consensus on pre-versus sub-pectoral implant positioning in this setting (q6f and g, appendix figure E1).

### 3.4 Special considerations: research and outcomes

**Fig. 5:**
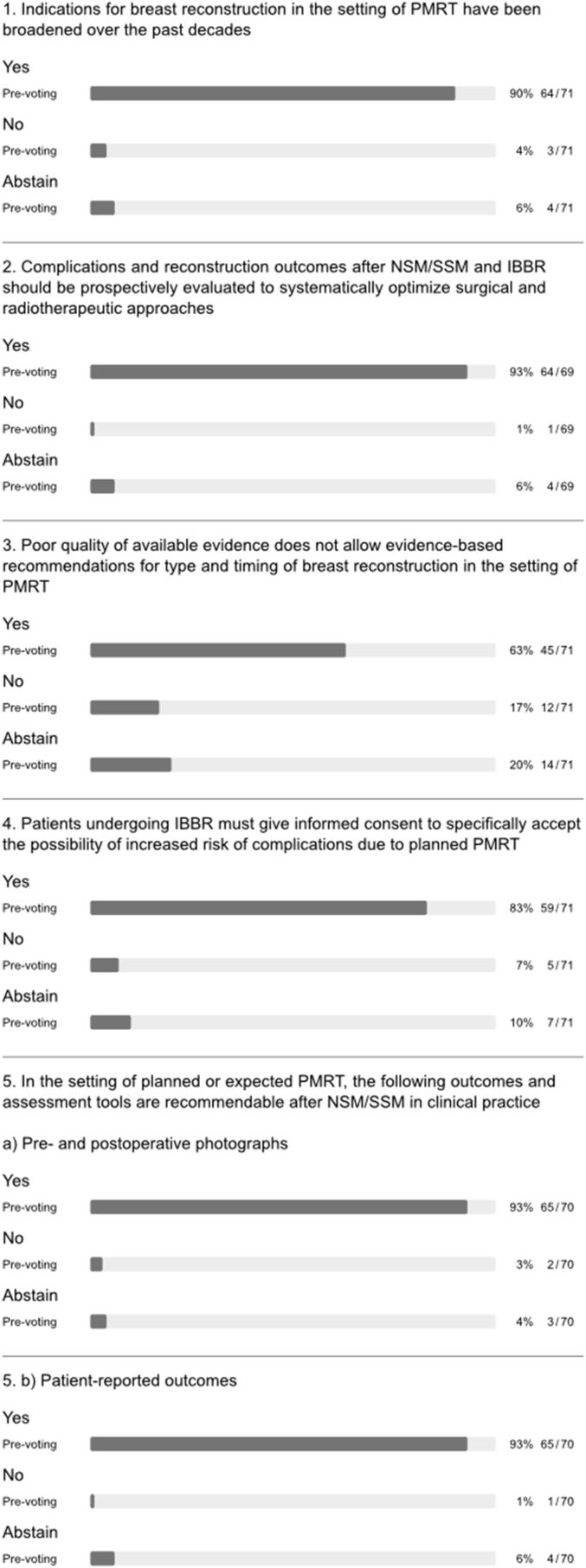
Special considerations.

Almost all panellists acknowledged current trends toward increasing use of BR in the setting of PMRT (q1, Fig. 5) [10]. The panel endorsed the need for prospective studies to optimize surgical and radiation treatments and conceded that the poor quality of available data broadly precludes evidence-based recommendations at this time (q2 and 3, Fig. 5). Of note, the OPBC ranked the question on the optimal type of reconstruction in the setting of planned adjuvant radiotherapy as top knowledge gap in the field already during the 2019 consensus conference [15]. A randomized controlled trial (RCT) design, as suggested by the scientific secretaries at the time, achieved not even a majority recommendation by the panel during two rounds of voting. It was considered not appropriate mostly due to a lack of feasibility. The study design was then adjusted according to the panel discussion into a prospective cohort study with propensity score matching and patient-reported satisfaction with breast, assessed by the BREAST-Q questionnaire at two years, as primary outcome. The question on the optimal timing of reconstruction in the setting of planned adjuvant radiotherapy was ranked as second most important priority in 2019. Therefore, the study design was adjusted and the panel finally achieved consensus to recommend a prospective registry to commonly address type and timing and the present project to focus on this important topic. This year, the OPBC voting results stressed the need for phase III RCTs to specifically address the optimal timing of implant-based BR, the positioning of implants and the use of adjunctive mesh. Of note, multiple observational studies over the past three years on pre-versus sub-pectoral implant-based BR have predominantly shown either no difference or marginally favoured pre-pectoral positioning [[24], [25], [26], [27], [28], [29], [30], [31], [32], [33]]. However, most were small, retrospective and single-centre studies, with only a few prospective or multicentre studies [25,26,28]. The OPBC-02/PREPEC trial is a pragmatic multicentre RCT designed to investigate QoL two years after pre-versus sub-pectoral implant-based BR and has currently randomized 245 of a total of 372 patients at 22 breast centres in 6 countries [34]. One of the formal substudies prospectively investigates the impact of pre-versus sub-pectoral implant-based BR on risk of early complications. Rates of unplanned reoperation were reported to be as high as 59% after immediate implant-based BR in the setting of PMRT [35]. Until risk profiles are better understood and strategies to reduce morbidity are optimized, the panel endorsed the viewpoint that patients undergoing implant-based BR must be fully informed and consent to the possibility of increased risk of complications in the setting of planned PMRT (q4, Fig. 5). Panellists and members could not agree on an acceptable upper limit for failure rate at two years after implant-based BR in daily practice (5% vs 10% vs 15%; q6, Fig. 1).

Almost all panellists supported use of pre- and postoperative photographs and prospective collection of patient-reported outcomes (q5a and b, Fig. 5). The majority of panellists and members sanctioned use of BREAST-Q either in entirety or selected scales for this purpose (q7, Fig. 1) [[36], [37], [38], [39], [40], [41]].

### 3.5 Post-mastectomy radiation therapy

**Fig. 6:**
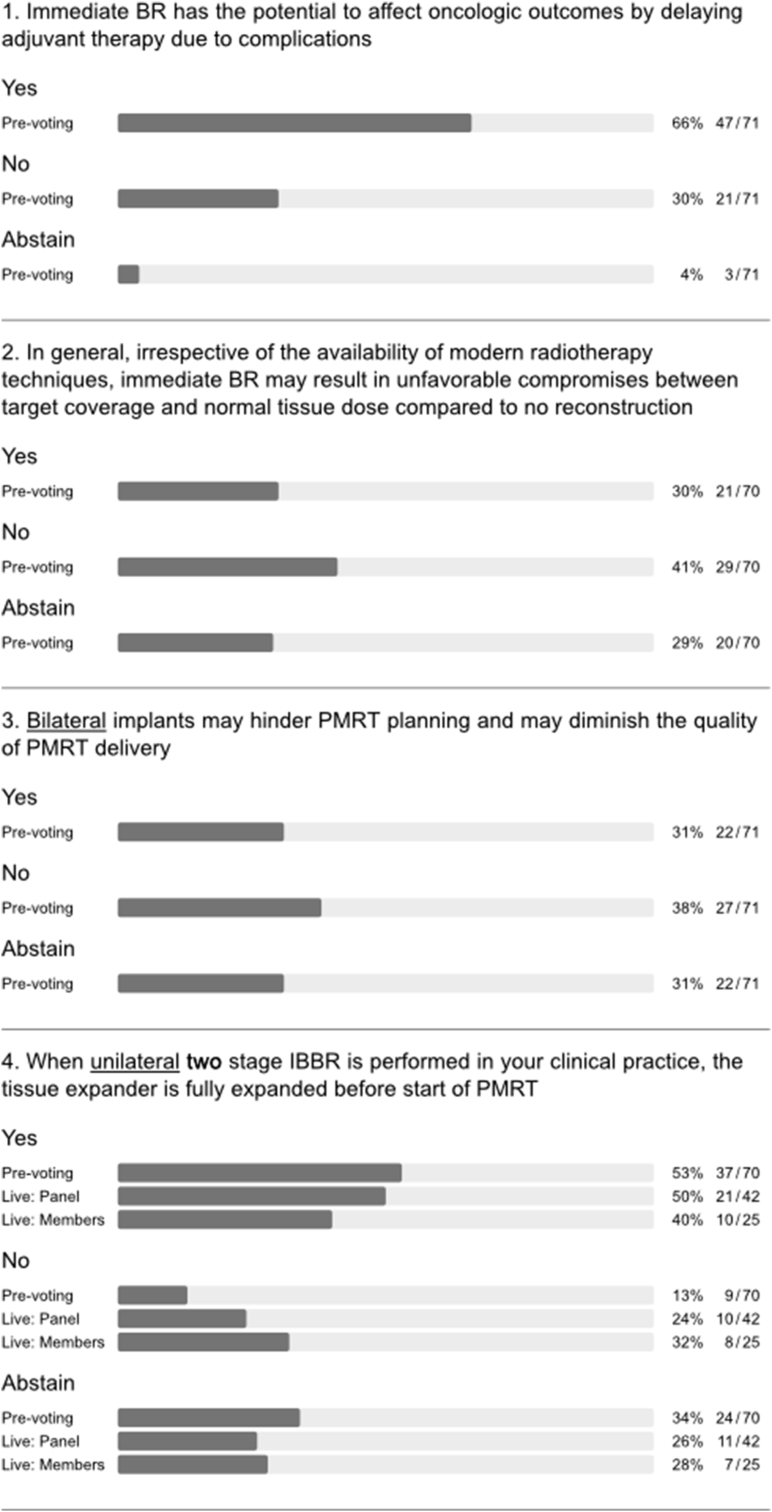

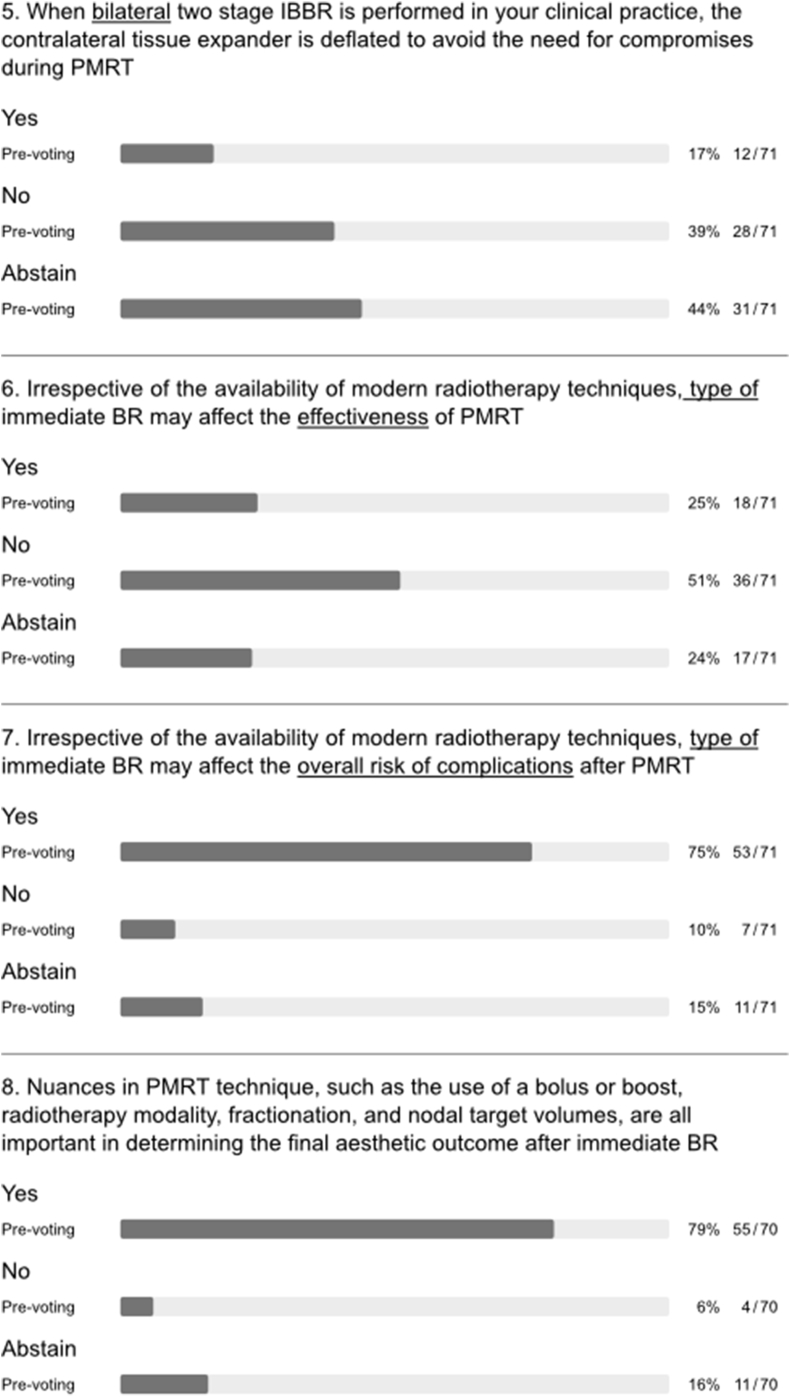
Post-mastectomy radiation therapy.

A majority of the panel felt that immediate BR has the potential to affect oncologic outcomes by delaying adjuvant therapy due to complications (q 1, Fig. 6). Clinical studies are inconsistent in reports of how postoperative complications affect recurrence and survival in patients undergoing immediate BR [[42], [43], [44], [45]]. Indeed, one of the largest studies showed that patients with postoperative complications had significantly worse disease-free survival than those without complications (hazard ratio (HR) 2.25; P = 0.015) [45]. However, this remained significant in patients who received adjuvant therapy without delay (8 weeks or less after surgery; HR 2.45; P = 0.034). After intense discussion of this topic, the question was re-phrased to ask whether immediate BR impairs oncologic outcomes by delaying adjuvant therapy in clinical practice. About half of panellists and members rejected that statement (q8, Fig. 1) and it was discussed that whilst there may be delays in some patients with potential impact on oncological safety, overall the average delay following PMBR is not clinically significant.

**Fig. 7:**
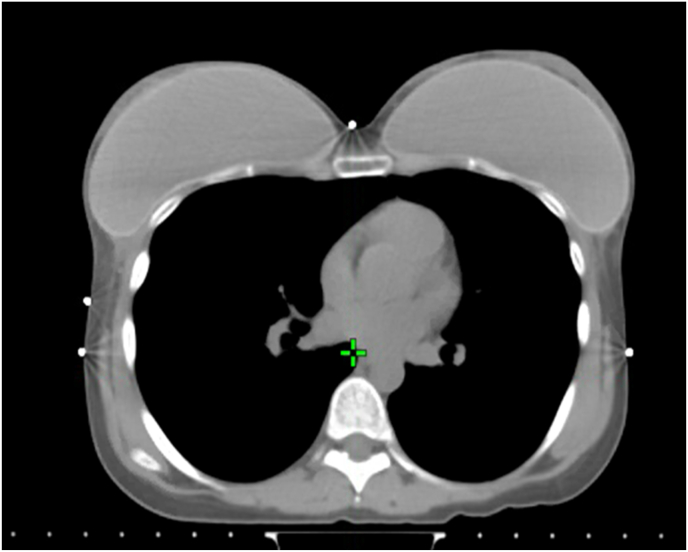
Post-mastectomy radiotherapy planning in patient with bilateral implant-based breast reconstruction.

There was major disagreement regarding whether immediate BR with creation of a breast mound compromised the accuracy of radiation dosimetry in terms of target coverage and normal tissue dose irrespective of modern radiotherapy techniques (q2, Fig. 6). Similarly, there was disagreement as to whether bilateral placement of implants impairs PMRT planning and quality of PMRT delivery (q3, Fig. 6). Indeed, early experience with immediate BR resulted in compromised target coverage and/or dose to organs at risk in case of PMRT. This was most apparent for irradiation of left-sided tumours, internal mammary nodes, and for cases of bilateral reconstruction [46]. Later reports suggested that correct target volume definition and modern radiation techniques can reduce the risks posed by BR, be this unilateral or bilateral [[47], [48], [49]]. To date, various measures can be applied to minimize dosage to organs at risk whilst ensuring adequate coverage of target volumes such as deep inspiration breath hold with or without continuous positive airway pressure (CPAP) [50,51]. Techniques for PMRT continue to evolve and routine use of a bolus for mastectomy cases is controversial as this may be associated with increased toxicity without improving local control [52]. Therefore, current European consensus guidelines do not recommend a bolus unless deemed necessary to ensure that the therapeutic dose of irradiation adequately covers those areas at high-risk for recurrence, e.g., in skin invading cancer [53]. Moreover, data on safety and efficacy in the setting of breast reconstruction is lacking [54]. Nonetheless, a boost in this setting was commonly practiced to enhance radiation dosage to the mastectomy scar in order to reduce local recurrence [55]. A study by Naoum et al. aimed to evaluate whether a chest wall boost was independently associated with reconstructive complications [55]. The study cohort included patients who had delayed reconstruction procedures. Scar boost was significantly linked with higher rates of infection, skin necrosis, and implant exposure. Furthermore, a boost dose was independently associated with a higher risk of complete implant failure and addition of a boost did not improve local tumor control, even among high-risk subgroups. Therefore, routine use of a boost or bolus for PMRT cases with or without reconstruction is not recommended. It is mandatory that radiation planning is tailored to the surgical procedure with awareness of potential adverse radiation effects on BR and adherence to international guidelines [53,[56], [57], [58]].

In contemporary practice, the type of BR is usually determined by body habitus, patient preference, and expertise of the surgeon. PMRT planning is rarely taken into account but close liaison between the surgical and radiation teams from the outset will facilitate optimal clinical decision-making in terms of BR and PMRT. In real-world practice, shape and size of the reconstructed breast mound can challenge PMRT planning and dose delivery (Fig. 7). Additionally, in case of expander with a metallic port, the ability to determine the accurate dose distribution and accurate RT delivery may be hindered [59].

Fig. 7: Axial view of radiation CT planning of a young patient who underwent bilateral mastectomy for left-sided breast cancer and immediate implant-based breast reconstruction. The size, shape and position of the reconstruction challenged the delivery of radiation to the left breast and regional lymphatics. Radiation is a trade-off between the objectives of target volume coverage and exposure of organs at risk. The radiation technique affects the interplay between these objectives (e.g., low dose bath to the lung, dose to the contralateral breast) but cannot escape the physical properties of the radiation beam.

Bearing in mind the impact of reconstructed breast volume on PRMT delivery, the panel also addressed the issue of volume in relation to tissue expanders. About half each of panellists and members opted for full expansion of the expander before PMRT in the case of unilateral two-stage BR. However, the others were divided between rejection and abstention. This reflected a degree of controversy and uncertainty (q4, Fig. 6), which was more apparent when asking whether the contralateral expander should be deflated after bilateral two-stage BR (q5, Fig. 6). From a radiation perspective, the volume of the expander at the time of CT planning and during irradiation should be maintained, as dosimetry is based on the target volume at the time of CT planning. Complete inflation can hinder PMRT planning and necessitate deflation of the expander prior to PMRT. Modern radiation techniques can ameliorate but not eliminate the physical properties of the radiation beam [60,61]. Use of volumetric-based PMRT and advanced radiation techniques to overcome a “non-anatomical” protruding reconstructed breast may result in unnecessary exposure of organs at risk and a low-dose-bath of radiation (leading to potential toxicity, late heart morbidity and risk of secondary cancers) [60,61]. Half of the panel rejected the statement that irrespective of the availability of modern radiotherapy techniques, type of immediate breast reconstruction may influence the effectiveness of PMRT (q 6, Fig. 6). However, there was consensus among panellists that the type of immediate BR affects overall risk of complications with PMRT, irrespective of modern radiotherapy, but PMRT techniques will impact upon final aesthetic outcome (q7 and q8, Fig. 6).

## 4 Conclusions

During the 2021 OPBC consensus conference, a large international panel comprised of breast surgery specialists, leading radiation oncologists and patient advocates was convened to systematically develop recommendations for mastectomy, BR and PMRT. The panel agreed that surgical technique for NSM/SSM should not be modified when PMRT is planned; it favoured the use of autologous over implant-based BR in the setting of PMRT due to lower long-term risk of complications and recommended immediate and delayed-immediate approaches. The panel strongly felt that PMRT is not an absolute contraindication for implant-based BR despite higher overall rates of complications. Nonetheless, no specific recommendations were made regarding implant positioning, use of mesh or timing due to absence of high-quality evidence to guide treatment. The panel encouraged routine use of pre- and postoperative photographs and endorsed patient-reported outcomes in clinical practice. It was acknowledged that shape and size of the reconstructed breast can be a geometric challenge for radiotherapy planning and the importance of PMRT techniques in determining the final aesthetic outcome after immediate BR was emphasized. Moreover, the panel unanimously supported the need for prospective studies, especially randomised trials, and proposed that surgical and radiation oncology teams work together at the outset to evaluate optimal sequencing and techniques for integrating PMRT with BR for each patient.

## Credit author statements

**Allweis**: Conceptualization, Data curation, Writing – review & editing. Barry: Conceptualization, Data curation, Writing – review & editing. **Benson**: Conceptualization, Data curation, Writing – review & editing. **Biazus**: Conceptualization, Data curation, Writing – review & editing. **Bjelic-Radisic**: Conceptualization, Data curation, Writing – review & editing. **Blohmer**: Conceptualization, Data curation, Writing – review & editing. **Bowles**: Conceptualization, Methodology, Validation, Data curation, Writing – review & editing. **Brenelli**: Conceptualization, Data curation, Writing – review & editing. Bucher: Conceptualization, Data curation, Writing – review & editing. **Cardoso**: Conceptualization, Data curation, Writing – review & editing. **Carmon**: Conceptualization, Data curation, Writing – review & editing. **Castrezana**: Conceptualization, Data curation, Writing – review & editing. **Catanuto**: Conceptualization, Data curation, Writing – review & editing. **de Boniface**: Conceptualization, Methodology, Software, Validation, Formal analysis, Investigation, Data curation, Writing – review & editing, Visualization, Project administration. **Dieroff Hay**: Conceptualization, Methodology, Validation, Data curation, Review & Editing. **Dubsky**: Conceptualization, Data curation, Writing – review & editing. **Elder**: Conceptualization, Data curation, Writing – review & editing. **El-Tamer**: Conceptualization, Data curation, Writing – review & editing. Fairbrother: Conceptualization, Data curation, Writing – review & editing. **Faridi**: Conceptualization, Data curation, Writing – review & editing. **Fitzal**: Conceptualization, Methodology, Validation, Data curation, Writing – review & editing. **Formenti**: Conceptualization, Data curation, Writing – review & editing. **French**: Conceptualization, Data curation, Writing – review & editing. **Galimberti**: Conceptualization, Data curation, Writing – review & editing**.Garcia-Etienne**: Conceptualization, Data curation, Writing – review & editing. **Gentilini**: Conceptualization, Data curation, Writing – review & editing. **Gnant**: Conceptualization, Data curation, Writing – review & editing. **Gonzalez**: Conceptualization, Data curation, Writing – review & editing. **Gouveia**: Conceptualization, Data curation, Writing – review & editing. **Gruber**: Data curation, Writing – review & editing. **Gulluoglu**: Conceptualization, Data curation, Writing – review & editing. **Günthert**: Conceptualization, Data curation, Writing – review & editing. **Hadar**: Conceptualization, Data curation, Writing – review & editing. **Harder**: Conceptualization, Data curation, Writing – review & editing. **Haug**: Conceptualization, Data curation, Writing – review & editing. Hauser: Conceptualization, Data curation, Writing – review & editing. **Heil**: Conceptualization, Methodology, Validation, Data curation, Writing – review & editing. **Hoffmann**: Conceptualization, Data curation, Writing – review & editing. **Joukainen**: Conceptualization, Data curation, Writing – review & editing. **Kaidar-Person**: Conceptualization, Data curation, Writing – review & editing. Kappos: Conceptualization, Data curation, Writing – review & editing. **Karadeniz Çakmak**: Conceptualization, Data curation, Writing – review & editing. **Karanlik**: Conceptualization, Data curation, Writing – review & editing. **Karhunen**-**Enckell**: Conceptualization, Data curation, Writing – review & editing. **Katapodi**: Conceptualization, Data curation, Writing – review & editing. **Kauer-Dorner**: Conceptualization, Data curation, Writing – review & editing. **Kauhanen**: Conceptualization, Data curation, Writing – review & editing. **King**: Conceptualization, Data curation, Writing – review & editing. **Knauer**: Conceptualization, Data curation, Writing – review & editing. **Kneser**: Conceptualization, Data curation, Writing – review & editing. **Koller**: Conceptualization, Data curation, Writing – review & editing. **Kontos**: Conceptualization, Data curation, Writing – review & editing. Koppert: Conceptualization, Data curation, Writing – review & editing. **Kovacs**: Conceptualization, Data curation, Writing – review & editing. **Krol**: Conceptualization, Data curation, Writing – review & editing. **Kuemmel**: Conceptualization, Data curation, Writing – review & editing. **Kühn**: Conceptualization, Data curation, Writing – review & editing. **Kurzeder**: Conceptualization, Data curation, Writing – review & editing. **Lagergren**: Conceptualization, Data curation, Writing – review & editing. **Letzkus**: Conceptualization, Data curation, Writing – review & editing. Maggi: Conceptualization, Data curation, Writing – review & editing. **Mátrai**: Conceptualization, Data curation, Writing – review & editing. **Montagna**: Conceptualization, Data curation, Writing – review & editing. Morrow: Conceptualization, Writing – review & editing. **Mucklow**: Conceptualization, Data curation, Writing – review & editing. **Paulinelli**: Conceptualization, Data curation, Writing – review & editing. **Poortmans**: Conceptualization, Methodology, Validation, Data curation, Writing – review & editing. **Potter**: Conceptualization, Data curation, Writing – review & editing. **Pusic**: Conceptualization, Data curation, Writing – review & editing. **Reitsamer**: Conceptualization, Data curation, Writing – review & editing. **Romics**: Conceptualization, Data curation, Writing – review & editing. Rubio: Conceptualization, Data curation, Writing – review & editing. **Rutgers**: Conceptualization, Data curation, Writing – review & editing. **Sacchini**: Conceptualization, Data curation, Writing – review & editing. **Saccilotto**: Conceptualization, Methodology, Software, Validation, Formal analysis, Investigation, Data curation, Writing – review & editing, Visualization, Project administration. **Schrenk**: Conceptualization, Data curation, Writing – review & editing. **Schwab**: Conceptualization, Data curation, Writing – review & editing. Sezer: Conceptualization, Data curation, Writing – review & editing. **Shaw**: Conceptualization, Methodology, Software, Validation, Formal analysis, Investigation, Data curation, Writing – review & editing, Visualization, Project administration. Steffens: Conceptualization, Data curation, Writing – review & editing. **Svensjö**; : Conceptualization, Data curation, Writing – review & editing. Tampaki: Conceptualization, Data curation, Writing – review & editing. **Tausch**: Conceptualization, Data curation, Writing – review & editing. **Tsoutsou**: Conceptualization, Data curation, Writing – review & editing. Urban: Conceptualization, Data curation, Writing – review & editing. **Vrancken Peeters**: Conceptualization, Data curation, Writing – review & editing. **Walker**: Conceptualization, Data curation, Writing – review & editing. **Weber**: Conceptualization, Methodology, Software, Validation, Formal analysis, Investigation, Resources, Data curation, Writing – original draft, Writing – review & editing, Visualization, Supervision, Project administration. **Wyld**: Conceptualization, Data curation, Writing – review & editing. **Zimmermann**: Conceptualization, Data curation, Writing – review & editing. **Zwahlen**: Conceptualization, Data curation, Writing – review & editing.

## Role of the funding source

This work was supported by the 10.13039/100008237Department of Surgery of the 10.13039/100016015University Hospital of Basel. The funding source had no role in study design; in the collection, analysis and interpretation of data; in the writing of the report; and in the decision to submit the article for publication. This research did not receive any specific grant from funding agencies in the public, commercial, or not-for-profit sectors.

## Declaration of competing interests

No competing interests in the current work were reported. The authors declare the following financial interests/personal relationships which may be considered as potential competing interests:

F. Brenelli had personal honoraria for Roche, MSD and Zodiac.

P. Dubsky received other from Amgen, AstraZeneca, Pfizer, Roche and Merck, grants from Cepheid/Danaher, Agendia, Myriad and from Oncomark.

M. Gnant reports personal fees/travel support from Amgen, DaiichiSankyo, AstraZeneca, EliLilly, LifeBrain, Nanostring, Novartis; an immediate family member is employed by Sandoz.

Support for meetings and teaching tasks has been paid to the research Department directed by Y. Harder from Establishment Labs, S.A, Costa Rica, Integra Life Sciences, USA and Hilotherm GmbH, Germany.

S Kuemmel has minority non-profit ownership at WSG Study Group; has a consulting/advisory board role at Amgen, AstraZeneca, Celgene, Daiichi-Sankyo, Genomic Health, Lilly, MSD, Novartis, Seagen. Pfizer, pfm Medical, Roche, Somatex, Seagen, Hologic; and received fees from Roche, Somatex, Novartis, Lilly, and personal fees from Roche, Novartis and Hologic.

Ch. Kurzeder receives honoraria from Tesaro, GSK, Astra Zeneca, Novartis, PharmaMar, Genomic Health, Roche, Eli Lilly S.A, Pfizer, Daichi, and travel fees from GSK, Astra Zeneca, Roche. He has a consulting or advisory role for Tesaro, GSK, Astra Zeneca, Novartis, PharmaMar, Genomic Health, Roche, Eli Lilly S.A, Merck MSD, Pfizer.

Travel, Accommodations, Expenses: GSK, Astra Zeneca, Roche.

A. Pusic is a co-developer of the BREAST-Q and receives royalties when it is used in for-profit, industry-sponsored clinical trials.

M. Walker has received personal honoraria from Guerbet and Roche Products Pty Ltd.

W. P. Weber received research from Takeda Pharmaceuticals International paid to the Swiss Group for Clinical Cancer Research (SAKK) and personal honoraria from Genomic Health, Inc, USA. for meetings was paid to his institution from Sandoz, Genomic Health, Medtronic Medtronic, Novartis Oncology, Pfizer and Eli Lilly.

S. Formenti reports: Consultant for: Bayer, Bristol Myers Squibb, Varian, ViewRay, Elekta, Janssen, Regeneron, GlaxoSmithKline, Eisai, Astra Zeneca, Merck US, EMD Serono/Merck, Genentech/ROCHE, Boheringer, Accuray.

Grant/Research support from: Bristol Myers Squibb, Varian, Regeneron, Merck, Celldex, ArcusM.Morrow reports personal fees from Exact Sciences and Roche.

All other authors declare no competing interests.
