# Supplementary material for: Oncoplastic breast consortium recommendations for mastectomy and whole breast reconstruction in the setting of post-mastectomy radiation therapy
Source: Breast. 2022 Mar 18;63:123–39. doi: 10.1016/j.breast.2022.03.008 (PMC8976143; doi:10.1016/j.breast.2022.03.008)

Supplementary Material (uploaded as single PDF file)

- Table of contents

- Appendix A: Program and protocol

- Appendix B.1: Expert representatives

- Appendix B.2: Staff

- Appendix B.3.1: 2021 OPBC panel by discipline and country

- Appendix B.3.2: Characteristics of 2021 OPBC panellists

- Appendix B.3.3. Characteristics of 2021 OPBC member attendance with live voting

- Appendix C: Pre-voting questionnaire for 2021 OPBC consensus conference

- Appendix D: Questionnaires to assess characteristics of OPBC panelists

- Appendix figure E.1: Supplementary results of pre-voting and live voting

- Appendix F: Graphical (visual) abstract

Supplementary appendix A: Program and protocol

Program for OPBC Consensus Conference 2021 on September 02 from 1pm to 5pm (CET)

TIME (CET)	*Session*	*Speaker*	*Moderator*	*Discussants*
Strategies for mastectomy and whole breast reconstruction in the setting of post-mastectomy radiotherapy				
13.00 – 13.10	***Introduction and Welcome***	Walter P. Weber		
13.10 – 13.25	***View of the plastic surgeon***	Andrea Pusic	Jörg Heil	
13.25 – 13.40	***Discussion***		Jörg Heil	Susanne Dieroff Hay<br>Michael Gnant
13.40 – 13.55	***View of the oncoplastic surgeon***	Jana de Boniface	Zoltan Matrai	
13.55 – 14.10	***Discussion***		Zoltan Matrai	Kimberly Bowles<br>Cicero Urban
14.10 – 14.25	***View of the radiation oncologist***	Philip Poortmans	Florian Fitzal	
14.25 – 14.40	***Discussion***		Florian Fitzal	Maria Katapodi<br>Lynda Wyld
**14.40 – 16.50**	*OPBC Consensus Confere*	Walter P. Weber<br>Jana de Boniface		
16.50 – 17.00	***Wrap up and Conference close***	Walter P. Weber<br>Jana de Boniface		

Protocol

OPBC expert panel 2021

The Oncoplastic Breast Consortium (OPBC) was founded in March 2017 as global non-profit organization and currently consists of 544 breast surgeons and 38 patient advocates from 79 countries. The OPBC is committed to bringing safe and effective oncoplastic breast surgery to routine patient care, namely oncoplastic breast conserving surgery (OPS), nipple-sparing (NSM) and skin-sparing mastectomy (SSM) with immediate breast reconstruction and aesthetic flat closure after conventional mastectomy. The global OPBC expert panel consists of 82 oncologic, oncoplastic and plastic breast surgeons from private, public, community and academic settings in 22 countries selected by evident expertise in breast cancer management with a practice primarily dedicated to breast cancer. In addition, the panel includes eight patients from five countries with longtime experience and established international reputation as patient advocates. Finally, the 2021 OPBC panel further contains six radiation oncologists who were invited based on scientific achievement and international standing.

Selection of topic

The 2018 OPBC consensus conference revealed major heterogeneity in whole breast reconstruction practice after NSM/SSM when radiotherapy is planned, and a majority of the panel agreed that there is a need for standardization of type and timing of reconstruction in the setting of adjuvant radiotherapy (Breast Cancer Res Treat. 2018 Dec;172(3):523-537.). The 2019 OPBC consensus conference ranked the type and timing of reconstruction in the setting of adjuvant radiotherapy as the two most important of a total of 38 knowledge gaps in the field (Lancet Oncol. 2020 Aug;21(8):e375-e385.)

Aim

The OPBC plans to address relevant questions about type and timing of mastectomy and whole breast reconstruction with planned radiotherapy and provide expert panel consensus recommendations that define best OPBC practices regarding indications, contraindications, surgical technique and outcome assessment.

Development of questionnaire

The predefined protocol of the conference will be published on the OPBC website and continuously updated. The identification of the questions for the conference will follow this pre-specified protocol: All relevant questions that have been addressed during the OPBC 2018 conference on NSM/SSM and immediate reconstruction will be asked again to assess changes over time based on new evidence that became available in the meantime. The two co-chairs will add key questions to the list based on their expert opinion. This preliminary set of questions will be further refined by the OPBC study group and

two dedicated patient advocates based on a specific literature search (see below). Thereafter, the list will be sent to the entire OPBC community (544 breast surgeons and 38 patient advocates from 79 countries) and the six panel radiation oncologists to give feedback, as well as report additional questions. The organizers will adjust the questions according to feedback from the OPBC community and refine the list by iterative consultation with the panelists over the months preceding the conference.

We will purposefully refrain from using a systematic literature search as basis for questionnaire development because we want the OPBC to identify and address questions that are relevant in clinical practice irrespective of available evidence to inform treatment. In support of questionnaire development, however, two members of staff (Elisabeth Kappos and Nadia Maggi) will independently perform an specific search in PubMed, MEDLINE, Embase and the Cochrane Central Register of Controlled Trials (CENTRAL) from 2000-2021 (search terms "mastectomy, subcutaneous" OR "mastectomy" AND "subcutaneous" OR "subcutaneous mastectomy" OR "nipple" AND "sparing" AND "mastectomy" OR "nipple sparing mastectomy" OR “breast reconstruction” OR whole-breast reconstruction” OR “breast reconstructive surgery” OR autologous breast reconstruction” OR “implant-based breast reconstruction” OR “post-mastectomy radiotherapy OR “irradiation” OR “radiotherapy” OR “breast reconstruction algorithm” OR “PMRT reconstruction” OR “PMRT breast reconstruction” OR “breast reconstruction algorithm radiation”). Their review of all abstracts and full texts of relevant articles will be used to finalize the questionnaire and help to prepare the chairs and moderators for the consensus conference. After the conference, it will be used as basis to write the manuscript.

Pre-voting on all questions will be performed prior to conference on Sept 02 for three reasons: 1. To serve as back-up in case of technical failure during live voting, 2. To provide opportunity to participate for panelists who cannot attend live voting, 3. To provide opportunity for all expert representatives to review the voting results on August 16 and define the exact voting agenda for the consensus conference. Live voting during the consensus conference may cover all questions again or focus on specific questions where no consensus was reached at pre-voting or where voting results should be endorsed. Results of pre-voting will be shown to panel and audience for the first time during conference on Sept 02 to allow spontaneous discussion by panelists. Two members of staff (Nadia Maggi and Fabienne Schwab) will document the discussion in written, which will also be recorded.

There will be three types of questions: Firstly, the OPBC will address prevalent questions in clinical practice with or without expected controversy to assess their relevance and seek consensus on the impact of post-mastectomy radiotherapy on various aspects of mastectomy and breast reconstruction; secondly, the OPBC will address questions in the field of PMRT -outside of the specific expertise of most panelists- to evaluate the opinion and knowledge of the OPBC expert panel in this closely related

field; thirdly -and most importantly- the OPBC will address relevant questions to guide clinical practice for mastectomy and breast reconstruction in the context of post-mastectomy radiotherapy.

Timelines:

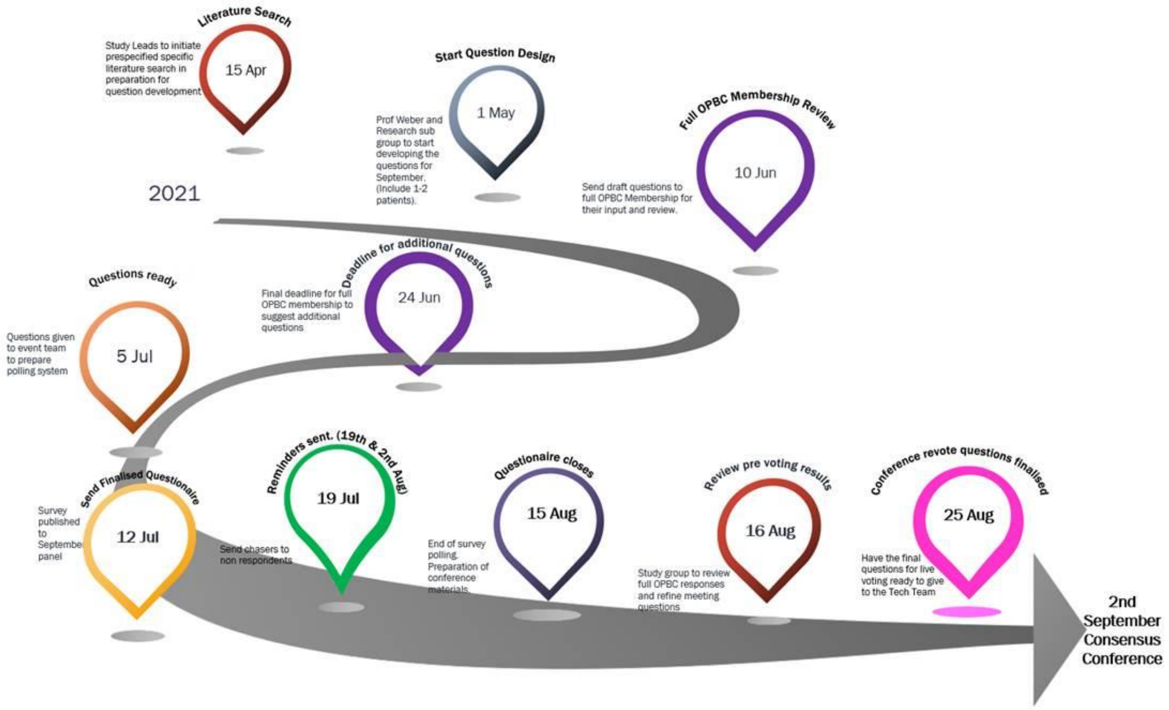

Consensus conference

The 2021 OPBC consensus conference on 02 September 2021 will be held fully virtually for the first time. During the meeting, three panel members will present their view as plastic surgeon (Andrea Pusic), oncoplastic surgeon (Jana de Boniface) and radiation oncologist (Philip Poortmans), followed by an interactive discussion using Pigeonhole Live technology. In the second half, the questions and corresponding results of pre-voting will be presented by pre-specified panelists, followed by live voting on selected questions to the extent technically feasible by the OPBC panelists and members who are present during the consensus conference. Results of live voting will be displayed by OPBC panelists versus members to facilitate discussion. Re-voting will be performed whenever indicated and technically feasible.

For most statements or questions, voting will be in the format yes, no or abstain, but for a minority, the single most appropriate answer will be selected from the list of options. Simple majority will be defined by agreement among 51-75% of the panelists and consensus by agreement above 75%. Abstaining will be recommended if panel members have conflict of interest or feel that the question was not clear or outside of their expertise, or that the correct answer was missing.

Report

The questions, answers and discussions will be brought into context with current evidence from the literature in the form of this report. For this purpose, the specific literature search that was performed for development of the questionnaire will be considered by the chairs and expert representatives, who selectively include additional references cited in those publications and articles that will be identified through searches of their own files. The report will be circulated among all 96 panelists in an iterative process until agreement will be reached on the wording for each question, which will convey the strength of panel support for each recommendation. Voting results will be shown graphically and as exact numbers.

Protocol originally published on OPBC website on 08 June 2021

Protocol amendments

02 July 2021:

Consensus conference

In the second half, the questions and corresponding results of pre-voting will be presented by pre-specified panelists, followed by live voting on selected questions to the extent technically feasible by the OPBC panelists and members who are present during the consensus conference. Results of live voting will be displayed by OPBC panelists versus members to facilitate discussion. Re-voting will be performed whenever indicated and technically feasible.

Appendix B.1: Expert representatives

Jana de Boniface (co-chair), MD, Walter Paul Weber (co-chair), MD, Kimberly Bowles (patient advocate), Susanne Dieroff Hay (patient advocate), Zoltan Matrai, MD, Florian Fitzal, MD, Jörg Heil, MD

Appendix B.2: Staff

Nadia Maggi, MD, Elisabeth Kappos, MD, Fabienne Schwab, MD, Liliana Castrezana, MD, Orit Kaidar-Person, MD, Daniel Steffens, MD, Janna Krol, MD

Appendix B.3.1: 2021 OPBC panel by discipline and country

Name/Surname	Discipline	Country
Eduardo Gonzalez	Surgeon	Argentina
Elisabeth Elder	Surgeon	Australia
James French	Surgeon	Australia
Melanie Walker	Surgeon	Australia
Florian Fitzal	Surgeon	Austria
Michael Gnant	Surgeon	Austria
Daniela Kauer-Dorner	Radiation Oncologist	Austria
Rupert Koller	Surgeon	Austria
Roland Reitsamer	Surgeon	Austria
Peter Schrenk	Surgeon	Austria
Philip Poortmans	Radiation Oncologist	Belgium
Jorge Biazus	Surgeon	Brazil
Fabricio Brenelli	Surgeon	Brazil
Regis Paulinelli	Surgeon	Brazil
Cicero Urban	Surgeon	Brazil
Jaime Letzkus	Surgeon	Chile
Tibor Kovacs	Surgeon	China
Sarianna Joukainen	Surgeon	Finland
Susanna Kauhanen	Surgeon	Finland
Ulla Karhunen-Enckell	Surgeon	Finland
Vesna Bjelic-Radisic	Surgeon	Germany
Jens-Uwe Blohmer	Surgeon	Germany
Andree Faridi	Surgeon	Germany
Jörg Heil	Surgeon	Germany
Juergen Hoffmann	Surgeon	Germany
Ulrich Kneser	Surgeon	Germany
Sherko Kuemmel	Surgeon	Germany
Thorsten Kühn	Surgeon	Germany
Michalis Kontos	Surgeon	Greece
Ekaterini Christina Tampaki	Surgeon	Greece
Zoltán Mátrai	Surgeon	Hungary
Mitchel Barry	Surgeon	Ireland

Name/Surname	Discipline	Country
Tanir Allweis	Surgeon	Israel
Moshe Carmon	Surgeon	Israel
Tal Hadar	Surgeon	Israel
Cecconi Agnese	Radiation Oncologist	Italy
Giuseppe Catanuto	Surgeon	Italy
Viviana Galimberti	Surgeon	Italy
Carlos A. Garcia-Etienne	Surgeon	Italy
Oreste Davide Gentilini	Surgeon	Italy
Linetta Koppert	Surgeon	Netherlands
Emiel Rutgers	Surgeon	Netherlands
Marie Jeanne Vrancken Peeters	Surgeon	Netherlands
Maria-Joao Cardoso	Surgeon	Portugal
Pedro Gouveia	Surgeon	Portugal
Isabel T Rubio	Surgeon	Spain
Jana de Boniface	Surgeon	Sweden
Susanne Dieroff Hay	Patient Advocate	Sweden
Jakob Lagergren	Surgeon	Sweden
Tor Svensjö	Surgeon	Sweden
Susanne Bucher	Surgeon	Switzerland
Peter Dubsky	Surgeon	Switzerland
Guenther Gruber	Radiation Oncologist	Switzerland
Andreas Günthert	Surgeon	Switzerland
Yves Harder	Surgeon	Switzerland
Martin Haug	Surgeon	Switzerland
Nik Hauser	Surgeon	Switzerland
Maria Katapodi	Patient Advocate	Switzerland
Michael Knauer	Surgeon	Switzerland
Christian Kurzeder	Surgeon	Switzerland
Rosine Mucklow	Patient Advocate	Switzerland
Jane Shaw	Patient Advocate	Switzerland
Christoph Tausch	Surgeon	Switzerland
Paula Tsoutsou	Radiation Oncologist	Switzerland
Walter P Weber	Surgeon	Switzerland
Frank Zimmermann	Radiation Oncologist	Switzerland
Daniel Rudolf Zwahlen	Radiation Oncologist	Switzerland
Bahadir M Gulluoglu	Surgeon	Turkey
Güldeniz Karadeniz Çakmak	Surgeon	Turkey
Hasan Karanlik	Surgeon	Turkey
Atakan Sezer	Surgeon	Turkey
Patricia Fairbrother	Patient Advocate	United Kingdom
Shelley Potter	Surgeon	United Kingdom
Laszlo Romics	Surgeon	United Kingdom
Lynda Wyld	Surgeon	United Kingdom
Kimberly Bowles	Patient Advocate	United States of America
Mahmoud El-Tamer	Surgeon	United States of America
Silvia Formenti	Radiation Oncologist	United States of America

Name/Surname	Discipline	Country
Tari King	Surgeon	United States of America
Giacomo Montagna	Surgeon	United States of America
Monica Morrow	Surgeon	United States of America
Andrea Pusic	Surgeon	United States of America
Virgilio Sacchini	Surgeon	United States of America

Appendix B.3.2: Characteristics of 2021 OPBC panellists

**Surgeons**	**n = 69**	
**Board certificate**		
Surgery	42	
Gynecology	16	
Plastic surgery	11	
**Gender**		
Female	20	
Male	49	
**Years of experience** (mean)	21	(SD 8.06)
**Estimated number of breast surgery procedures performed in 2020**		
0-20	1	
21-50	4	
51-100	17	
>100	46	
**Type of breast centre**		
Academic	49	
Public	12	
Private	8	
**Patient Advocates**	**n = 6**	
**Type of surgery**		
Breast conserving surgery	1	
Mastectomy without reconstruction	2	
Mastectomy with implant-based reconstruction	1	
Mastectomy with autologeous reconstruction	2	

Radiation Oncologists	n = 9	
**Gender**		
Female	3	
Male	6	
Years of experience (mean)	26	(SD 7.88)
**Estimated number of breast cancer treatments performed in 2020**		
>100	8	
**Type of breast center**		
Academic	5	
Public	3	
Private	1	
**Total**	**n = 83**	

Appendix B.3.3. Characteristics of 2021 OPBC member attendance with live voting

Total	n = 52	
**Medical Professionals**	**n = 48**	
Breast Surgeons	38	
Gynaecologists	8	
Plastic surgeon	1	
Radiation oncologist	1	
**Gender**		
Female	22	
Male	26	
Years of experience (mean)	13.19	(SD 8.1)
**Estimated number of patients with breast cancer treated in 2020**		
0-20	2	
21-50	7	
51-100	14	
>100	25	

**Type of breast centre**	
Acedemic	25
Public	15
Private	8
**Attendance by continent**	
Africa	3
Asia	9
Australia	2
Europe	33
North America	1
**Patient Advocates**	**n = 4**
**Type of surgery**	
Mastectomy without reconstruction	1
Mastectomy with implant-based reconstruction	2
Mastectomy with autologeous reconstruction	1
**Attendance by continent**	
Europe	3
North America	1

Appendix C: Pre-voting questionnaire for 2021 OPBC consensus conference

Abbreviations used in questionnaire: NSM (nipple-sparing mastectomy), PMRT (post-mastectomy radiotherapy), BR (breast reconstruction), IBBR (implant-based breast reconstruction)

Nipple-/ skin-sparing mastectomy (NSM/SSM)

1. **Planned or expected PMRT is a contraindication to nipple preservation** *(vote with yes, no or abstain)*

2. **Planned or expected PMRT may have an impact on the choice of incision for NSM** *(vote with yes, no or abstain)*

3. **In a woman with cup size ≥ C and ptosis ≥ grade 2 and planned or expected PMRT, but no other obvious risk factors for nipple necrosis and no signs of ischemia during surgery, would you be willing to offer NSM with use of** *(vote separately for a-d with yes, no or abstain)*
   a. Skin reduction and nipple-areola pedicles **independently from** breast reconstruction technique
   b. Skin reduction and nipple-areola pedicles **only** when autologous breast reconstruction is planned
   c. Skin reduction and free nipple grafting
   d. Without skin reduction

4. **In the setting of planned or expected PMRT, NSM should be performed less radically in terms of conservation of anatomic structures and thickness of skin and nipple flaps** *(vote with yes, no or abstain)*

5. **PMRT can be associated with clinically relevant hypopigmentation of the nipple-areola complex and reduction of areola diameter** *(vote with yes, no or abstain)*

Type of breast reconstruction

6. **PMRT increases the overall risk of complications (defined as an adverse postoperative, surgery-related event requiring additional treatment) after all types of IBBR (one stage, two stage, pre-pectoral, sub-pectoral, with synthetic mesh, with biologic mesh, without mesh)** (vote with yes, no or abstain)

7. **PMRT increases the overall risk of complications (defined as an adverse postoperative, surgery-related event requiring additional treatment) after the following type of autologous reconstruction** (vote with yes, no or abstain for a-c)

a. **Immediate autologous reconstruction**
b. **Immediate autologous reconstruction combined with implant**
c. **Delayed-immediate autologous reconstruction: first surgery (expander or implant)**
d. **Delayed-immediate autologous reconstruction: second surgery (autologous reconstruction)**
e. **Delayed autologous reconstruction**

8. **Among patients who are expected to receive PMRT, the overall risk of complications associated with immediate autologous reconstruction compared to IBBR is** *(please choose one of the following answers)*
    a. Higher
    b. Lower
    c. Comparable
    d. Abstain

9. **Outside of clinical trials, planned or expected PMRT is a contraindication to** *(vote separately for a-h with yes, no or abstain)*
    a. All types of immediate breast reconstruction
    b. Immediate autologous breast reconstruction
    c. Immediate autologous breast reconstruction combined with an implant/expander
    d. Immediate one-stage **sub-**pectoral IBBR with a biologic or synthetic mesh
    e. Immediate one-stage **pre-**pectoral IBBR with a biologic or synthetic mesh
    f. Immediate one-stage **pre-**pectoral IBBR without a biologic or synthetic mesh
    g. Two-stage IBBR (sub-pectoral expander to definitive implant)
    h. Delayed-immediate breast reconstruction (expander/implant to autologous reconstruction)

10. **In case of expected PMRT and planned autologous reconstruction, your preferred method - provided that patient preference and anatomical preconditions are met- is** *(please choose one of the following answers)*
    a. Immediate autologous reconstruction
    b. Immediate reconstruction as combination of an implant and a flap
    c. Delayed-immediate reconstruction (expander/implant to autologous reconstruction after PMRT)
    d. Delayed autologous reconstruction after PMRT
    e. Abstain

11. **In case of expected PMRT and planned <u>IBBR</u>, your preferred method -provided that patient preference and anatomical preconditions are met- is** *(please choose one of the following answers)*
   a. Immediate one-stage **pre**-pectoral IBBR without synthetic or biologic mesh
   b. Immediate one-stage **sub**-pectoral IBBR without synthetic or biologic mesh
   c. Immediate one-stage **pre**-pectoral IBBR with synthetic mesh
   d. Immediate one-stage **sub**-pectoral IBBR with synthetic mesh
   e. Immediate one-stage **pre**-pectoral IBBR with biologic mesh
   f. immediate one-stage **sub**-pectoral IBBR with biologic mesh
   g. Two-stage IBBR (pre- or sub-pectoral expander to definitive implant, with or without use of any mesh at any stage)
   h. Abstain

12. **In the setting of PMRT, pre-pectoral IBBR is associated with higher risk of complications and failure rates than sub-pectoral IBBR** <u>(please choose yes, no or abstain)</u>

13. **Which of the following types of reconstruction do you recommend -provided that patient preference and anatomical preconditions are met- to achieve the <u>lowest overall risk of complications</u> when PMRT is expected** *(please choose one of the following answers)*

   a. Immediate autologous reconstruction
   b. Delayed-immediate reconstruction (expander/implant to autologous reconstruction after PMRT)
   c. Delayed autologous reconstruction after PMRT
   d. Immediate reconstruction with combination of an implant and a flap
   e. Immediate one-stage **pre**-pectoral IBBR without synthetic or biologic mesh
   f. Immediate one-stage **sub**-pectoral IBBR without synthetic or biologic mesh
   g. Immediate one-stage **pre**-pectoral IBBR with synthetic mesh
   h. Immediate one-stage **sub**-pectoral IBBR with synthetic mesh
   i. Immediate one-stage **pre**-pectoral IBBR with biologic mesh
   j. Immediate one-stage **sub**-pectoral IBBR with biologic mesh
   k. Two-stage IBBR (pre- or sub-pectoral expander to definitive implant, with or without use of mesh at any stage) with irradiation of expander
   l. Two-stage IBBR (pre- or sub-pectoral expander to definitive implant, with or without use of any mesh at any stage) with irradiation of final implant
   m. Abstain

14. **Which of the following types of reconstruction do you recommend -provided that patient preference and anatomical preconditions are met- to achieve the <u>best aesthetic results</u> when PMRT is planned or expected** *(please choose one of the following answers)*

    a. Immediate autologous reconstruction
    b. Delayed-immediate reconstruction (expander/implant to autologous reconstruction after PMRT)
    c. Delayed autologous reconstruction after PMRT
    d. Immediate reconstruction with combination of an implant and a flap
    e. Immediate one-stage **pre**-pectoral IBBR without synthetic or biologic mesh
    f. Immediate one-stage **sub**-pectoral IBBR without synthetic or biologic mesh
    g. Immediate one-stage **pre**-pectoral IBBR with synthetic mesh
    h. Immediate one-stage **sub-**pectoral IBBR with synthetic mesh
    i. Immediate one-stage **pre**-pectoral IBBR with biologic mesh
    j. Immediate one-stage **sub-**pectoral IBBR with biologic mesh
    k. Two-stage IBBR (pre- or sub-pectoral expander to definitive implant, with or without use of any mesh at any stage) with irradiation of expander
    l. Two-stage IBBR (pre- or sub-pectoral expander to definitive implant, with or without use of any mesh at any stage) with irradiation of final implant
    m. Abstain

Timing of breast reconstruction

15. **Optimal timing of delayed autologous reconstruction in women with rapid skin healing following PMRT** *(please choose one of the following answers)*
    a. A minimum of 12 months after end of PMRT
    b. A minimum of 6 months after end of PMRT
    c. A minimum of 3 months after end of PMRT
    d. ≤3 months after end of PMRT
    e. Abstain

16. **In your clinical practice, are there established indications for delayed IBBR after PMRT?** *(please vote yes, no or abstain)*

17. **If you voted yes to the previous question (all others please abstain): Which strategies do you recommend to reduce complications after IBBR following PMRT** (please vote with yes, no or abstain for each one)
    a. Highly cohesive implants

b. Nanotextured implants
c. Polyurethane implants
d. Use of synthetic mesh
e. Use of biologic mesh
f. Pre-pectoral IBBR
g. Sub-pectoral IBBR
h. Fat grafting

18. **Optimal timing of two stage IBBR in women receiving PMRT <u>without</u> adjuvant chemotherapy** *(please choose one of the following answers)*
    a. Irradiation of tissue expanders
    b. Irradiation of permanent implants

19. **Optimal timing of two stage IBBR in women receiving PMRT <u>with</u> adjuvant chemotherapy** *(please choose one of the following answers)*
    a. Irradiation of tissue expanders
    b. Irradiation of permanent implants

20. **Optimal timing of change to implant after PMRT to tissue expander in women with rapid skin healing following PMRT** *(please choose one of the following answers)*
    a. A minimum of 12 months after end of PMRT
    b. A minimum of 6 months after end of PMRT
    c. A minimum of 3 months after end of PMRT
    d. ≤3 months after end of PMRT
    e. Abstain

21. **In your clinical practice, are there established indications for the use of neoadjuvant radiotherapy before mastectomy and immediate BR?** (vote with yes, no or abstain)

Special considerations

22. **Indications for breast reconstruction in the setting of PMRT have been broadened over the past decades** (vote with yes, no or abstain)

23. **Do you recommend fat grafting to address contour deformities or volume deficiency at any time point during or after NSM/SSM and immediate <u>autologous</u> BR followed by PMRT?** (please vote with yes, no or abstain)

24. **Do you recommend fat grafting to address contour deformities, implant rippling or volume deficiency at any time point during or after NSM/SSM and immediate IBBR followed by PMRT?** (please vote with yes, no or abstain)

25. **If you voted yes to the previous question (all others abstain): Optimal timing of fat grafting after NSM/SSM and immediate IBBR followed by PMRT?** (*please choose one of the following answers*)
    a. A minimum of 12 months after end of PMRT
    b. A minimum of 6 months after end of PMRT
    c. A minimum of 3 months after end of PMRT
    d. ≤3 months after end of PMRT
    e. Abstain

26. **Poor quality of available evidence does not allow evidence-based recommendations for type and timing of breast reconstruction in the setting of PMRT** (please vote with yes, no or abstain)

27. **Complications and reconstruction outcomes after NSM/SSM and IBBR should be prospectively evaluated to systematically optimize surgical and radiotherapeutic approaches** (please vote with yes, no or abstain)

28. **Patients undergoing IBBR must give informed consent to specifically accept the possibility of increased risk of complications due to planned PMRT** *(please vote yes, no or abstain)*

29. **Nuances in PMRT technique, such as the use of a bolus or boost, radiotherapy modality, fractionation, and nodal target volumes, are all important in determining the final aesthetic outcome after immediate BR** (please vote with yes, no or abstain)

30. **In the setting of planned or expected PMRT, the following outcomes and assessment tools are recommendable after NSM/SSM in clinical practice** *(vote separately for a-e with yes, no or abstain)*
    a. Pre- and postoperative photographs
    b. Patient-reported outcomes
    c. All or selected scales of BREAST-Q
    d. All or selected scales of EORTC QLQ-BRECON-23
    e. All or selected scales of BRECON-31

31. **In the setting of planned or expected PMRT, which of the following measures do you recommend most strongly for use in all future studies that involve patient-reported outcomes?** *(please choose one of the following answers)*
    a. All or selected scales of BREAST-Q
    b. All or selected scales of EORTC QLQ-BRECON-23
    c. All or selected scales of BRECON-31
    d. None of the above
    e. Abstain

Post-mastectomy radiotherapy

The final set of questions assesses the opinion and knowledge of the 2021 OPBC panel on the impact of immediate BR on delivery, safety and effectiveness of PMRT.

32. **Immediate BR has the potential to affect oncologic outcomes by delaying adjuvant therapy due to complications** (vote with yes, no or abstain)

33. **In general, irrespective of the availability of modern radiotherapy techniques, immediate BR may result in unfavorable compromises between target coverage and normal tissue dose compared to no reconstruction** (vote with yes, no or abstain)

34. **Irrespective of the availability of modern radiotherapy techniques, type of immediate BR may affect the <u>effectiveness</u> of PMRT** (vote with yes, no or abstain)

35. **Irrespective of the availability of modern radiotherapy techniques, type of immediate BR may affect the <u>overall risk of complications</u> after PMRT** (vote with yes, no or abstain)

36. **When <u>unilateral</u> one stage IBBR is performed in your clinical practice, the tissue expander is fully expanded before start of PMRT** *(please vote yes, no or abstain)*

37. **<u>Bilateral</u> implants may hinder PMRT planning and may diminish the quality of PMRT delivery** (vote with yes, no or abstain)

38. **When <u>bilateral</u> two stage IBBR is performed in your clinical practice, the contralateral tissue expander is deflated to avoid the need for compromises during PMRT** (vote with yes, no or abstain)

Appendix D: Questionnaires to assess characteristics of OPBC panelists

ONCOPLASTIC BREAST CONSORTIUM

Consensus conference on breast reconstruction with planned radiotherapy

PERSONAL INFORMATION FORM

PATIENT ADVOCATES

Name (optional): ____

Middle Name (optional): ____

Surname (optional): ____

Affiliation (if applicable): ____

Gender: Female ☐ Male ☐

Year of diagnosis: ____

Surgical procedure:

☐ Breast conserving surgery

☐ Mastectomy without reconstruction

☐ Mastectomy with implant-based reconstruction

☐ Mastectomy with reconstruction using your own body tissue

☐ No surgical treatment

☐ I prefer not to disclose this information

Consensus conference on breast reconstruction with planned radiotherapy

PERSONAL INFORMATION FORM

SURGEONS

Name: ____

Middle Name: ____

Surname: ____

Affiliation: ____

____

____

Board Certificate: **General Surgery** ☐ **Gynecology** ☐ **Plastic Surgery** ☐

Years of Experience: ____

Estimated Number of Breast Surgery Procedures Performed or Assisted in 2020:

0-20 ☐ 20-50 ☐ 50-100 ☐ 100+ ☐

Gender: Female ☐ Male ☐

Type of Breast Center: Academic ☐ Public ☐ Private ☐

2021 Consensus conference on breast reconstruction with planned radiotherapy

PERSONAL INFORMATION FORM

Radiation Oncologists

Name: ____

Middle Name: ____

Surname: ____

Affiliation: ____

____

____

Years of Experience: ____

Estimated number of patients with breast cancer treated in 2020:

0-20 ☐ 20-50 ☐ 50-100 ☐ 100+ ☐

Gender: Female ☐ Male ☐

Type of Breast Center: Academic ☐ Public ☐ Private ☐ Not applicable ☐

Appendix figure E.1: Supplementary results of pre-voting and live voting

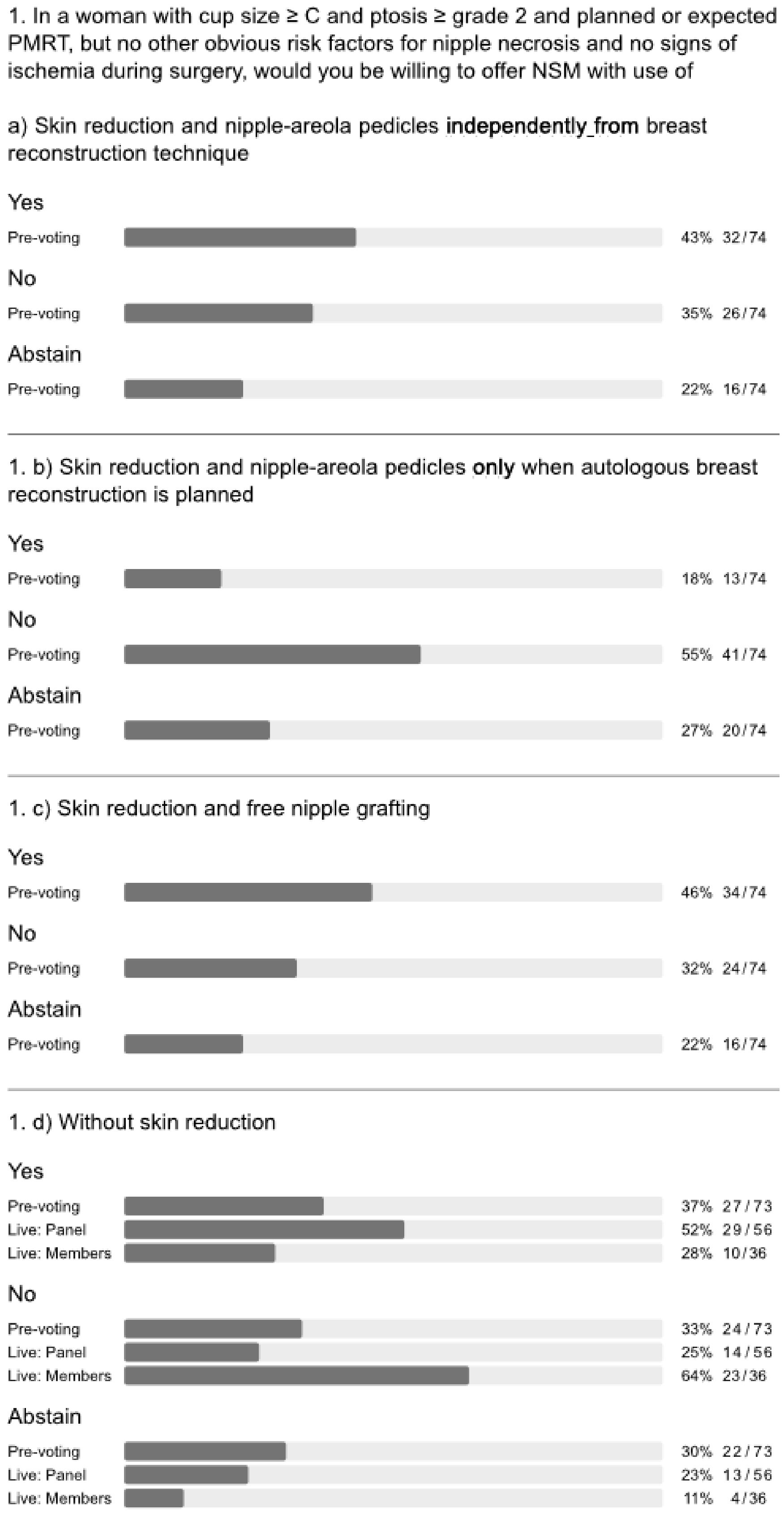

2. PMRT increases the overall risk of complications – defined as an adverse postoperative, surgery-related event requiring additional treatment – after the following type of <u>autologous</u> reconstruction

a) Immediate autologous reconstruction

Yes	Pre-voting	66%	46 / 70
No	Pre-voting	23%	16 / 70
Abstain	Pre-voting	11%	8 / 70

---

2. b) Immediate autologous reconstruction combined with implant

Yes	Pre-voting	88%	60 / 68
No	Pre-voting	4%	3 / 68
Abstain	Pre-voting	7%	5 / 68

---

2. c) Delayed-immediate autologous reconstruction: first surgery (expander or implant)

Yes	Pre-voting	75%	52 / 69
No	Pre-voting	19%	13 / 69
Abstain	Pre-voting	6%	4 / 69

---

2. d) Delayed-immediate autologous reconstruction: second surgery (autologous reconstruction)

Yes	Pre-voting	49%	34 / 69
No	Pre-voting	38%	26 / 69
Abstain	Pre-voting	13%	9 / 69

---

2. e) Delayed autologous reconstruction

Yes	Pre-voting	51%	35 / 69
No	Pre-voting	41%	28 / 69
Abstain	Pre-voting	9%	6 / 69

3. Outside of clinical trials, planned or expected PMRT is a contraindication to

a) All types of immediate breast reconstruction

Yes	Pre-voting	4%	3/70
No	Pre-voting	93%	65/70
Abstain	Pre-voting	3%	2/70

3. b) Immediate autologous breast reconstruction

Yes	Pre-voting	3%	2/70
No	Pre-voting	90%	63/70
Abstain	Pre-voting	7%	5/70

3. c) Immediate autologous breast reconstruction combined with an implant/ expander

Yes	Pre-voting	9%	6/69
No	Pre-voting	84%	58/69
Abstain	Pre-voting	7%	5/69

3. d) Immediate one-stage **sub**-pectoral IBBR with a biologic or synthetic mesh

Yes	Pre-voting	11%	8/70
No	Pre-voting	76%	53/70
Abstain	Pre-voting	13%	9/70

3. e) Immediate one-stage **pre**-pectoral IBBR <u>with</u> a biologic or synthetic mesh

Yes	Pre-voting	17%	12 / 70
No	Pre-voting	70%	49 / 70
Abstain	Pre-voting	13%	9 / 70

---

3. f) Immediate one-stage **pre**-pectoral IBBR <u>without</u> a biologic or synthetic mesh

Yes	Pre-voting	20%	14 / 70
No	Pre-voting	71%	50 / 70
Abstain	Pre-voting	9%	6 / 70

---

3. g) Two-stage IBBR (sub-pectoral expander to definitive implant)

Yes	Pre-voting	6%	4 / 69
No	Pre-voting	88%	61 / 69
Abstain	Pre-voting	6%	4 / 69

---

3. h) Delayed-immediate breast reconstruction (expander/implant to autologous reconstruction)

Yes	Pre-voting	3%	2 / 70
No	Pre-voting	91%	64 / 70
Abstain	Pre-voting	6%	4 / 70

4. Which of the following types of reconstruction do you recommend –provided that patient preference and anatomical preconditions are met – to achieve the lowest overall risk of complications when PMRT is expected

Option		Percentage	Votes
Immediate autologous reconstruction	Pre-voting	16%	11 / 68
Delayed-immediate reconstruction (expander/implant to autologous reconstruction after PMRT)	Pre-voting	21%	14 / 68
Delayed autologous reconstruction after PMRT	Pre-voting	29%	20 / 68
Immediate reconstruction with combination of an implant and a flap	Pre-voting	0%	0 / 68
Immediate one-stage **pre**-pectoral IBBR without synthetic or biologic mesh	Pre-voting	3%	2 / 68
Immediate one-stage **sub**-pectoral IBBR without synthetic or biologic mesh	Pre-voting	3%	2 / 68
Immediate one-stage **pre**-pectoral IBBR with synthetic mesh	Pre-voting	4%	3 / 68
Immediate one-stage **sub**-pectoral IBBR with synthetic mesh	Pre-voting	0%	0 / 68
Immediate one-stage **pre**-pectoral IBBR with biologic mesh	Pre-voting	1%	1 / 68
Immediate one-stage **sub**-pectoral IBBR with biologic mesh	Pre-voting	1%	1 / 68
Two-stage IBBR (pre- or sub-pectoral expander to definitive implant, with or without use of mesh at any stage) with irradiation of expander	Pre-voting	3%	2 / 68
Two-stage IBBR (pre- or sub-pectoral expander to definitive implant, with or without use of any mesh at any stage) with irradiation of final implant	Pre-voting	1%	1 / 68
Abstain	Pre-voting	16%	11 / 68

5. Which of the following types of reconstruction do you recommend –provided that patient preference and anatomical preconditions are met – to achieve the best aesthetic results when PMRT is planned or expected

Immediate autologous reconstruction

Pre-voting 21% 15/70

Delayed-immediate reconstruction (expander/implant to autologous reconstruction after PMRT)

Pre-voting 40% 28/70

Delayed autologous reconstruction after PMRT

Pre-voting 16% 11/70

Immediate reconstruction with combination of an implant and a flap

Pre-voting 1% 1/70

Immediate one-stage **pre**-pectoral IBBR without synthetic or biologic mesh

Pre-voting 3% 2/70

Immediate one-stage **sub**-pectoral IBBR without synthetic or biological mesh

Pre-voting 1% 1/70

Immediate one-stage **pre**-pectoral IBBR with synthetic mesh

Pre-voting 3% 2/70

Immediate one-stage **sub**-pectoral IBBR with synthetic mesh

Pre-voting 0% 0/70

Immediate one-stage **pre**-pectoral IBBR with biologic mesh

Pre-voting 0% 0/70

Immediate one-stage **sub**-pectoral IBBR with biologic mesh

Pre-voting 1% 1/70

Two-stage IBBR (pre- or sub-pectoral expander to definitive implant, with or without use of mesh at any stage) with irradiation of expander

Pre-voting 4% 3/70

Two-stage IBBR (pre- or sub-pectoral expander to definitive implant, with or without use of any mesh at any stage) with irradiation of final implant

Pre-voting 0% 0/70

Abstain

Pre-voting 9% 6/70

6. Which strategies do you recommend to reduce complications after IBBR following PMRT if you voted Yes to the question if there established indications for delayed IBBR after PMRT

a) Highly cohesive implants

Yes	Pre-voting	32%	7 / 22
No	Pre-voting	68%	15 / 22

6. b) Nanotextured implants

Yes	Pre-voting	24%	5 / 21
No	Pre-voting	76%	16 / 21

6. c) Polyurethane implants

Yes	Pre-voting	33%	7 / 21
No	Pre-voting	67%	14 / 21

6. d) Use of synthetic mesh

Yes	Pre-voting	30%	6 / 20
No	Pre-voting	70%	14 / 20

6. e) Use of biologic mesh

Yes	Pre-voting	62%	13 / 21
No	Pre-voting	38%	8 / 21

6. f) Pre-pectoral IBBR

Yes	Pre-voting	53%	9 / 17
No	Pre-voting	47%	8 / 17

6. g) Sub-pectoral IBBR

Yes	Pre-voting	44%	8 / 18
No	Pre-voting	56%	10 / 18

6. h) Fat grafting

Yes	Pre-voting	73%	16 / 22
No	Pre-voting	27%	6 / 22

Appendix F: Graphical (visual) abstract

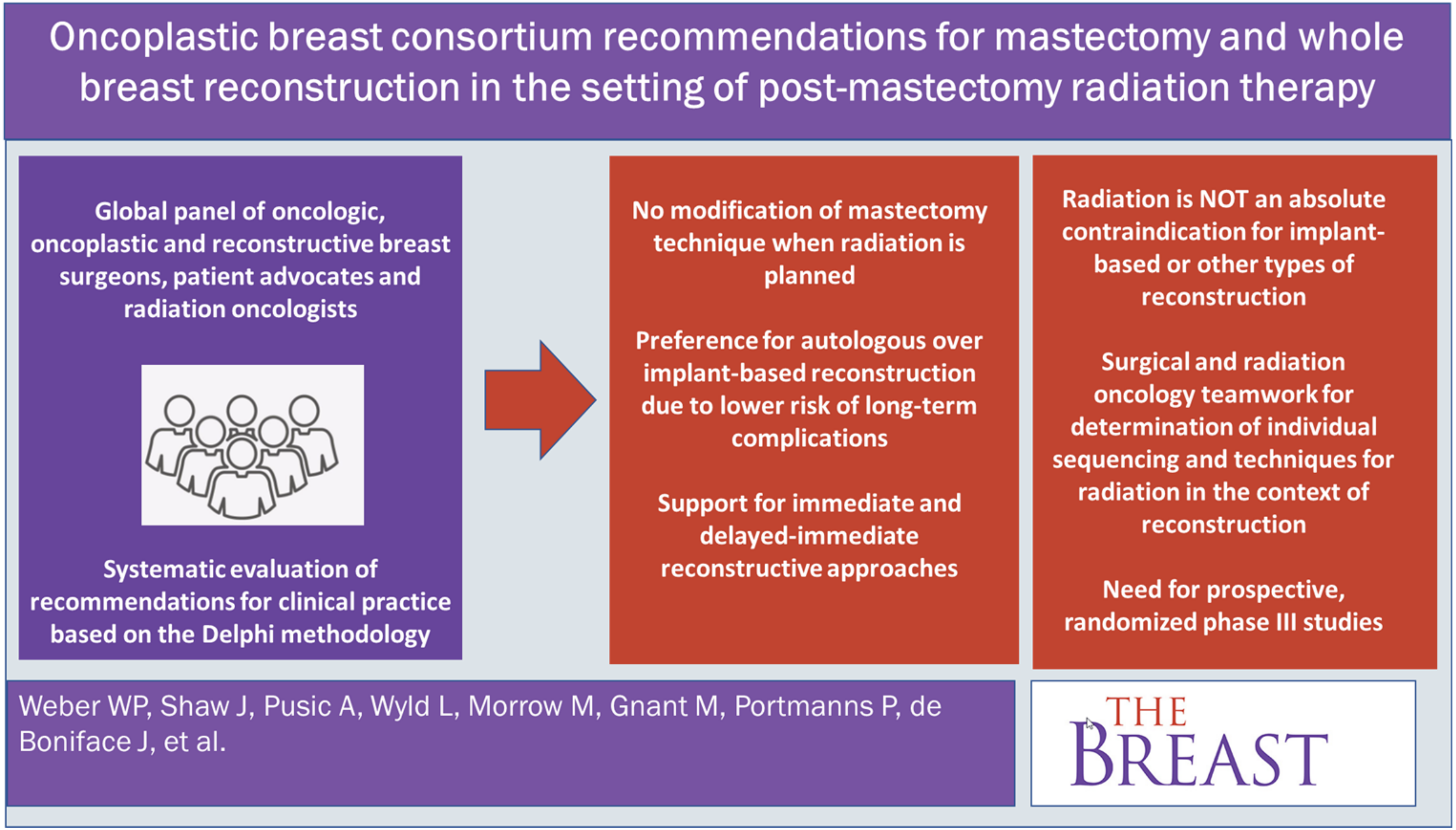

Supplement: Multimedia component 1 [file mmc1.pdf]
