# Supplementary material for: Oncoplastic breast consortium recommendations for mastectomy and whole breast reconstruction in the setting of post-mastectomy radiation therapy
Source: Breast. 2022 Mar 18;63:123–39. doi: 10.1016/j.breast.2022.03.008 (PMC8976143; doi:10.1016/j.breast.2022.03.008)
Supplement: Multimedia component 2 [file mmc2.pptx]

## Slide 1
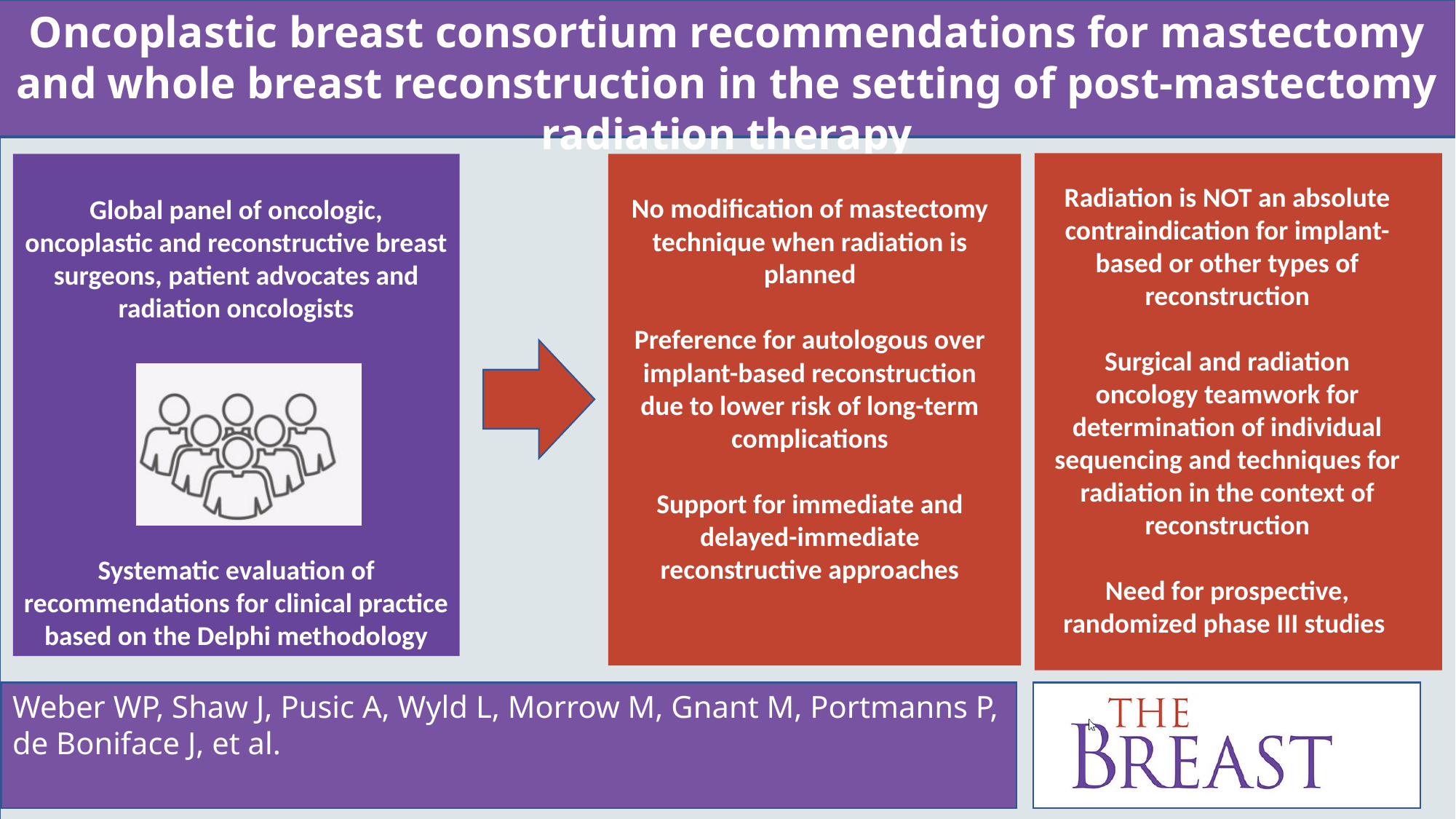

Oncoplastic breast consortium recommendations for mastectomy and whole breast reconstruction in the setting of post-mastectomy radiation therapy
Global panel of oncologic, oncoplastic and reconstructive breast surgeons, patient advocates and radiation oncologists
Systematic evaluation of recommendations for clinical practice based on the Delphi methodology
Radiation is NOT an absolute contraindication for implant-based or other types of reconstruction
Surgical and radiation oncology teamwork for determination of individual sequencing and techniques for radiation in the context of reconstruction
Need for prospective, randomized phase III studies
No modification of mastectomy technique when radiation is planned
Preference for autologous over implant-based reconstruction due to lower risk of long-term complications
Support for immediate and delayed-immediate reconstructive approaches
Weber WP, Shaw J, Pusic A, Wyld L, Morrow M, Gnant M, Portmanns P, de Boniface J, et al.
